# Spatial and phenotypic heterogeneity of resident and monocyte-derived macrophages during inflammatory exacerbations leading to pulmonary fibrosis

**DOI:** 10.3389/fimmu.2024.1425466

**Published:** 2024-07-19

**Authors:** Philip J. Moos, Jenna R. Cheminant, Sophie Cowman, Jessica Noll, Qiuming Wang, Teresa Musci, Alessandro Venosa

**Affiliations:** Department of Pharmacology and Toxicology, University of Utah College of Pharmacy, Salt Lake City, UT, United States

**Keywords:** alveolar type-2 cell, surfactant protein-C I73T mutant, pulmonary fibrosis, alveolar macrophages, monocyte-derived macrophages, apolipoprotein-E, fibronectin1, osteopontin1

## Abstract

**Introduction:**

Genetic mutations in critical nodes of pulmonary epithelial function are linked to the pathogenesis of pulmonary fibrosis (PF) and other interstitial lung diseases. The slow progression of these pathologies is often intermitted and accelerated by acute exacerbations, complex non-resolving cycles of inflammation and parenchymal damage, resulting in lung function decline and death. Excess monocyte mobilization during the initial phase of an acute exacerbation, and their long-term persistence in the lung, is linked to poor disease outcome.

**Methods:**

The present work leverages a clinical idiopathic PF dataset and a murine model of acute inflammatory exacerbations triggered by mutation in the alveolar type-2 cell-restricted Surfactant Protein-C [SP-C] gene to spatially and phenotypically define monocyte/macrophage changes in the fibrosing lung.

**Results:**

SP-C mutation triggered heterogeneous CD68^+^ macrophage activation, with highly active peri-injured cells relative to those sampled from fully remodeled and healthy regions. Ingenuity pathway analysis of sorted CD11b^-^SigF^+^CD11c^+^ alveolar macrophages defined asynchronous activation of extracellular matrix re-organization, cellular mobilization, and Apolipoprotein E (*Apoe)* signaling in the fibrosing lung. Cell-cell communication analysis of single cell sequencing datasets predicted pro-fibrogenic signaling (*fibronectin/Fn1, osteopontin/Spp1*, and *Tgfb1*) emanating from *Trem2/TREM2* ^+^ interstitial macrophages. These cells also produced a distinct lipid signature from alveolar macrophages and monocytes, characterized by *Apoe* expression. Mono- and di-allelic genetic deletion of ApoE in SP-C mutant mice had limited impact on inflammation and mortality up to 42 day after injury.

**Discussion:**

Together, these results provide a detailed spatio-temporal picture of resident, interstitial, and monocyte-derived macrophages during SP-C induced inflammatory exacerbations and end-stage clinical PF, and propose ApoE as a biomarker to identify activated macrophages involved in tissue remodeling.

## Introduction

Acute inflammatory exacerbations represent a key feature in the evolution of interstitial lung diseases (ILD). This cluster of chronic progressive pathologies includes idiopathic pulmonary fibrosis, non-specific interstitial pneumonia, connective tissue disease associated ILD, chronic hypersensitivity pneumonitis, pneumoconiosis, sarcoidosis, and more (1, 2). The exact sequence of events igniting these flare-ups have yet to be fully understood. However, epidemiological and experimental evidence suggest that a combination of genetic susceptibility, preexistent pulmonary and systemic conditions, biological aging/senescence of the alveolar compartment, and external stressors (environmental toxic exposure) contribute to the repeated cycles of focal inflammation, spatially heterogenous injury, and aberrant repair characteristic of fibrotic disease (3–7).

To date, over 60 mutations in the alveolar epithelial type 2 specific gene encoding for the surfactant protein C (SP-C) have been linked to the development of a fibrotic phenotype, with the isoleucine-to-threonine missense substitution at position 73 of the SP-C proprotein (SP-C^I73T^) representing the most common (8–10). Our group has previously characterized endoplasmic reticulum toxicity and macroautophagy block resulting from aberrant processing and trafficking of the SP-C proprotein and described the sequelae of immunological events accompanying tissue remodeling (8, 9, 11, 12). Here, we expand on this prior investigation by defining the spatial and phenotypic distribution of monocytes and macrophages responding to the initial epithelial injury and establish patterns of intercellular communication among cellular species in the lung.

Mounting evidence supports the notion that inflammatory monocyte mobilization in the fibrosing lung represents a valid indicator of poor disease prognosis (13, 14). Yet, experimental modeling and clinical trials designed to non-specifically target inflammation (e.g., corticosteroids, broad spectrum cytokine modulation) have revealed low efficacy or even harmful toxicities (15). Among the reasons for this therapeutic failure is the absence of a nuance approach capable of controlling the maturation, activation, and persistence of ontologically and phenotypically heterogeneous cellular entities, and the relatively fragmented understanding of the spatial distribution of cells and signals in a temporally extended pathology such as fibrosis (16–18). Experimental evidence highlights distinct transcriptomes emanating from tissue-resident and monocyte-derived macrophages (19, 20), with the latter generating a complex fibrogenic signature (10, 21, 22). The fibronectin/FN1, osteopontin/SPP1, tumor growth factor (TGF)β1, and interleukin 4/13 signaling pathways represent the most studied networks mediating tissue remodeling (23–27), while metabolic networks have gained traction as potential targets in chronic inflammatory diseases (28, 29). *In vitro* systems establish a reliance on glycolysis in response to canonical pro-inflammatory signals (IFNγ, LPS), juxtaposed to fatty acid oxidation, tricarboxylic acid cycle, and mitochondrial oxidative phosphorylation following challenge with anti-inflammatory and pro-remodeling signals (IL-10, IL-4/13, TGFβ1) (30–32). This evidence emphasizes the importance of factors governing lipid synthesis, handling, and metabolism (*PPARγ, LXR, FXR, SREBP1*) in regulating macrophage function (33, 34). The cholesterol and phospholipid transporter apolipoprotein E (ApoE) has been linked to monocyte-derived macrophage activation in chemical-induced injury, though there is limited evidence that these responses are consistent across the spectrum of fibrosis (35, 36).

Through a combination of bulk, single cell, and spatial transcriptomics we show a hyperactive niche of macrophages surrounding fully remodeled lung regions during fibrogenic exacerbations triggered by mutant SP-C induction. Cellular annotation and communication analysis identify time-related changes in intercellular networks, with alveolar and interstitial/*Trem2* ^+^ macrophages and inflammatory monocytes responsible for *Spp1, Fn1*, and *Tgfβ1* pro-fibrotic signaling. Analysis of human idiopathic PF confirms the presence of an interstitial population responsible for pro-fibrotic signaling in the diseased lung. Our findings also highlight disease-related shifts in lipid transcriptional signatures and interstitial/*Trem2* ^+^ macrophages as the sole cellular cluster expressing ApoE. While genetic ablation of ApoE in SP-C^I73T^ induced injury did not significantly impact fibrotic disease outcome, our results pinpoint this molecule as a potential biomarker identifying fibrogenic myeloid populations. Together, these results reveal temporal and phenotypic heterogeneity in the macrophage compartment and implicates *Trem2* ^+^ interstitial macrophages and their monocytic precursors as viable targets for anti-fibrogenic therapy.

## Materials and methods

### Sex as a biological variable

These studies utilized both male and female mice.

### Murine model of SP-C^I73T^ induced lung injury

Tamoxifen inducible SP-C^I73T^ mice were generated as previously reported (9). For studies investigating the role of the apoliproprotein E in SP-C^I73T^ induced injury, a parallel line was crossed with ApoE knock out mice purchased from Jackson laboratories (Strain #002052, The Jackson Laboratory, Bar Harbor, ME). Briefly, an estrogen receptor (ER)-2 controlled Flp-O recombinase strain knocked into the Rosa26 locus (The Jackson Laboratory). Adult homozygote SP-C^I73T^Flp mice (8-12 weeks) received two tamoxifen oral gavages three days apart (90 mg/kg each) to excise a neomycin cassette placed within the *Sftpc* gene. Both male and female animals were used for the studies. For studies involving apoE mutants, a SP-C^I73T^ApoE^WT/KO^ breeding pair was utilized (SP-C^I73T^ApoE^HET^) so as to generate wild type, heterozygous, and homozygous experimental littermates. Control groups mice are represented as pooled data from tamoxifen treated SP-C^I73T^ not expressing Flp-O recombinase or oil (vehicle) treated Flp-O expressing SP-C^I73T^ mice. All mice were housed under pathogen free conditions in AALAC approved barrier facilities at the Skaggs College of Pharmacy, University of Utah. All experiments were approved by the Institutional Animal Care and Use Committee, University of Utah.

### Reagents

Tamoxifen (non-pharmaceutical grade) was purchased from Sigma-Aldrich (St Louis, MO). Giemsa cytological stain was purchased from Sigma-Aldrich. Antibody list: *Spp1* (RNAscope^®^ Probe Green, Ref#435191); *Tgfb1* (RNAscope^®^ Probe Red, Ref# 407751-C2); *Apoe* (for *in situ* hybridization – Advanced Cell Diagnostics, RNAscope^®^ LS 2.5 Probe #313278; for immunohistochemical - Abcam; Cat #ab183597, 1:500; for western blot – Cell Signaling Technology, Cat #49285, 1:1000), CD68 for immunohistochemistry (Cell Signaling Technology; Cat # 97778; 1:1000). Flow cytometric panel for cell sorting and bulk sequencing of macrophages CD16/32 (clone 93; eBiosciences, San Diego, CA), CD11b (clone # M1/70; eFluo450, eBiosciences); Fixable Viability dye (Cat # 65-0865-14; eFluo780, eBiosciences); SigF (clone S17007L; PE-CF594, BD Biosciences, San Jose, CA); CD45 (clone 30-F11; PerCP5.5, Biolegend, San Diego, CA); CD11c (clone # N418; BV705, Biolegend); Ly6G (clone # 1A8; AF700, Biolegend); CD64 (clone X54-5/7.1; PE/Cy7, Biolegend); CD3 (clone # 17A2; BUV395, Biolegend). All other reagents were purchased from Thermo Fisher Scientific, Inc. (Waltham, MA) or Sigma-Aldrich.

### Bronchoalveolar lavage, cell counts, ELISA, and western blot analysis

Following terminal anesthesia, inert mice were subject to cannulation. Bronchoalveolar lavage (BAL) fluid was collected from mice using 1 mL sterile saline lavage and collected into a microcentrifuge tube. Four additional lavages were performed and collected in a separate container. The two fractions were then spun at 400 × g, 6 min. Supernatant from the first lavage was collected and immediately frozen at -80°C for ELISA and western blot analysis, while the two cellular pellets were combined and suspended in 1 ml of saline solution for cell counts, flow cytometric, or RNA sequencing analysis. BAL cells were enumerated using a NucleoCounter (New Brunswick Scientific, Edison, NJ). Aliquots of first lavage were analyzed for IL-4 and IL-13 levels using the Luminex platform (Panel MCYTOMAG-70K-17) following Thermo Fisher’s protocol. For western blot, equal volumes (15 µl) of thawed BAL fluid were loaded onto 4-12% NuPage Bis-Tris gels (ThermoFisher Scientific) with NuPage 4X LDS sample buffer (ThermoFisher Scientific) and then electrophoresed approximately 90 minutes using a constant voltage of 100V. Proteins from the gels were then transferred to a 0.45 μm PVDF membrane at 30V and 4°C for one hour and blocked in 5% non-fat dried milk (NFDM). The membranes were subsequently probed with primary ApoE antibodies. The SuperSignal West Dura Chemiluminescent Substrate detection system was applied before exposing the membrane on the ProteinSimple FluorChem M imager (BioTechne).

### Histology, histochemical and *In situ* hybridization analysis

For histological and histochemical analysis, lungs from unresponsive anesthetized mice were cleared of excess blood through cardiac perfusion with 0.9% sodium chloride solution. A 20-gauge cannula was inserted in the trachea for tissue fixation with 10% neutral buffer formalin at constant pressure (25 cm H_2_O). A suture was used to seal the tracheal opening upon cannula removal, thus avoiding pulmonary deflation during the fixation process. Tissue was placed in a histology cassette and submerged in 10% neutral buffer formalin for 72 h. The suture was then removed, and the lung sequentially moved to a 2% sucrose solution (in PBS, two washes of 5 min) and 70% ethyl alcohol. The submerged cassettes were submitted to the University of Utah histology core (Associated Regional and University Pathologists Inc.) for embedding. Paraffin blocks were sectioned at 6 µm thickness and used for Hematoxylin & Eosin (H&E) or immunohistochemical staining, alone or in combination with *in situ* hybridization as previously described (37). For protein staining, paraffin was removed using xylene solutions followed by gradient alcohol washes (100-50%). Citrate antigen retrieval (10.2 mM sodium citrate, pH 6.0, for 20 min) and endogenous peroxidase quenching with 3% hydrogen peroxide in methanol (30 min) were performed. Serum-based blocking (10% goat serum in PBS) preceded the overnight incubation with anti-rabbit primary antibody. In all studies, a serum/IgG control was used. During the second day, slides underwent incubation with a biotinylated secondary antibody (30 minutes, Vectastain Elite ABC kit, Vector Labs, Burlingame, CA) and chromogenic reaction achieved using a Peroxidase Substrate Kit DAB (Vector Labs). Counterstain was accomplished with Harris Modified Hematoxylin (Thermo Fisher Scientific, Inc.). For *in situ* hybridization studies, after paraffin removal with xylene/alcohol solutions, slides were air dried. Peroxidase quenching (10 min, away from light) was followed by antigen retrieval (20 min, RNAscope^®^ Target Retrieval Reagent, ACD) and incubation with protease IV (30min, RNAscope^®^ Protease IV Reagent, ACD). Excess solution was then washed off. Slides were then incubated for 2 hours in a 40°C hybridization oven with a *Spp1*, *Tgfb1, or Apoe* probe. A series of signal amplification steps (6 for single color detection, 10 for double-staining assay) and washes were followed by chromogen development. At this point the experiment was either concluded with counterstain and toluene-based permount coverslip placement, or the immunohistochemistry protocol resumed from the blocking and primary antibody step as described above.

### Fluorescence activated cell sorting

In some studies, following cardiac perfusion the left lobe was tied off with a suture and removed for flow cytometric and FACS analysis. Tissue was minced with surgical scissors and transferred into a 50 ml conical tube and incubated (37°C, 30 minutes) in DMEM + 5% FBS + 2 mg/ml Collagenase D (Cat #11088866001, Roche, Indianapolis, IN). Digested lungs were passed through 70-μm nylon mesh to obtain a single-cell suspension, counted and mixed with ACK Lysis Buffer (Thermo Fisher Scientific, Inc.) to remove any remaining red blood cells. The single cell suspension was counted and resuspended to yield 1 x 10^6^ cells per 100µl of flow cytometry staining buffer (PBS+0.1% sodium azide). Cells were then incubated with anti-mouse CD16/32 antibody for 10 min at 4°C to block nonspecific binding. This was followed by 30-minute incubation with fluorescently-tagged antibodies or appropriate isotype controls (0.25–1.5 µg/10^6^ cells) for 30 minutes (4°C). Cells were then spun and resuspended in staining buffer for viability staining (30 minutes at 4°C). Cells were fixed in 2% paraformaldehyde and sorted using a FACS ARIA (BD Biosciences). Alveolar macrophages (SigF^+^CD11b^-^CD11c^+^) were identified following forward and side scatter selection of singlet CD45^+^ viable cells. To ensure cell sorting of a purified population, a series of exclusion gates were designed to remove eosinophils (SigF^int^CD11b^+^CD11c^-^), neutrophils (Ly6G^+^) and lymphocytes (CD3^+^). All analysis was performed using FlowJo software (FlowJo, LLC, Ashland, Oregon).

### Bulk and single-cell RNA sequencing preparation and analysis

For bulk RNA sequencing studies (deposited on NCBI GEO GSE166300), sorted SigF^+^CD11b^-^CD11c^+^ macrophages underwent RNA extraction using Qiagen RNeasy Plus Universal mini kit following manufacturer’s instructions (Qiagen, Hilden, Germany). Extracted RNA samples were quantified using a Qubit 2.0 Fluorometer (Life Technologies, Carlsbad, CA, USA) and RNA integrity was checked using Agilent TapeStation 4200 (Agilent Technologies, Palo Alto, CA, USA). RNA sequencing libraries were prepared using the NEBNext Ultra RNA Library Prep Kit for Illumina following manufacturer’s instructions (NEB, Ipswich, MA, USA). Briefly, mRNAs were first enriched with Oligo(dT) beads. Enriched mRNAs were fragmented for 15 minutes at 94°C. First-strand and second strand cDNAs were subsequently synthesized. cDNA fragments were end-repaired and adenylated at 3’ends, and universal adapters were ligated to cDNA fragments, followed by index addition and library enrichment by limited-cycle PCR. The sequencing libraries were validated on the Agilent TapeStation (Agilent Technologies, Palo Alto, CA, USA), and quantified by using Qubit 2.0 Fluorometer (Invitrogen, Carlsbad, CA) as well as by quantitative PCR (KAPA Biosystems, Wilmington, MA, USA). The sequencing libraries were pooled and clustered on 1 lane of a flow cell. After clustering, the flowcell was loaded on the Illumina HiSeq4000 instrument according to manufacturer’s instructions. The samples were sequenced using a 2x150bp Paired End (PE) configuration. Image analysis and base calling were conducted by the HiSeq Control Software (HCS). Raw sequence data (bcl files) generated from Illumina HiSeq was converted into fastq files and de-multiplexed using Illumina’s bcl2fastq 2.17 software. One mismatch was allowed for index sequence identification. Analysis of RNA counts was performed using R (3.6.3) (38). Differential gene expression analysis was conducted using the hciR package (39). Further pathway analysis was conducted using IPA (QIAGEN Inc). Datasets were filtered using log2 fold change (minimum -1 or 1) and p-value cut offs (minimum p<0.05) to ensure an appropriate number of molecules (200-3000) were used in the IPA pipeline.

Single-cell RNA sequencing raw data of SP-C^I73T^ model were deposited in NCBI’s Gene Expression Omnibus and are accessible through GEO Series accession numbers GSE247520 and GSE196657. Tissue collection and single cell suspension were achieved using mechanical mincing, digestion in Collagenase D, red blood cell lysis, and suspension created by using a 70-μm strainer. RNA extraction and library preparation are described in the published manuscript (10). Mining of human control and IPF lungs are accessible through GSE136831 (40). As described by Adams et al., representative apical and basal segments of explanted lungs were minced mechanically, digested [elastase (30 U/ml) + deoxyribonuclease I (0.2 mg/ml) + liberase (0.3 mg/ml) + 1% penicillin/streptomycin], cleared of red blood cells, and single cell suspension created using a 100/70/40 strainers (40). Re-analysis of each single-cell dataset included dimension reduction and clustering by SCTransformation (0.3.5) using the Gamma-Poisson generalized linear model method (glmGamPoi, 1.8.0) and were performed using the Seurat (4.0.4) package (41–45). Multiple levels of resolution were evaluated using Clustree (0.5.0). The data was assessed for cell cycle effects using CellCycleScoring and regressed for uneven cell cycle expression across clusters. Cell types were identified using differential gene expression and all manual annotations were compared to those produced through automated classification using SingleR (1.10.0). A specific R package was used to interface with enrichR database (46). For pseudotime analysis, monocle-3 software was used, while cell-cell communication analysis was conducted with CellChat software (47–51).

### Spatial transcriptomic analysis

For spatial analysis (deposited on NCBI GEO GSE264128), neutral buffered formalin-fixed lungs were inflated without bronchoalveolar lavage. Paraffin-embedded sections were baked for 1 hour at 60°C and stained according to the NanoString Leica BOND RX RNA FFPE Semi-Automatic protocol. Following Proteinase K (1.0ug/mL, 15 min) and ER2 (20 min) processing, sections were incubated with fluorescently tagged antibodies against PanCytokeratin (PanCK, Novus Biologics, NBP2-33200AF488; 1:400), Syto83 (S11364; 1:10, Thermo Fisher Scientific, Inc.), CD45 (Nanostring Technologies, 121302304; 1:5), and CD68 (Abcam, ab125212; 1:25). Regions of interest (ROI; N = 3, healthy; N = 4, peri-injured, N = 5, injured/remodeled) were selected based on histopathological assessment of inflammation and epithelial thickening and remodeling. Tissue segmentation selectively identified CD45^+^CD68^+^ macrophages. The collection plate was then removed from the GeoMx instrument and prepared for sequencing. The GeoMx library was prepared, processed, and sequenced according to the NanoString NGS Readout User Manual Protocol (ref. MAN-10153-04). Sequencing was performed on the Illumina NovaSeq 6000, S2 v1.5 with a 100 cycles flowcell at 27 bp pair-end reads. Generated FASTQ files were then processed to DCC files utilizing the NanoString GeoMx NGS Pipeline according to manufacturer’s instructions. Gene expression was analyzed using Nanostring DSP analysis software (NanoString Technologies) with built-in statistical analyses. Raw data counts were run through internal quality control and biological probe quality control.

### Statistical analysis

Unless otherwise indicated, all data are presented as group mean ± SE. Statistical analyses were performed with Prism GraphPad 9.4 (GraphPad Software, San Diego, CA). Student’s t-tests were used for paired data; for analyses involving multiple groups, one-way or two-way analysis of variance (ANOVA) was performed with *post hoc* testing as indicated. Survival analyses were performed using Log Rank (Mantel-Cox) test. For spatial analysis, segments were filtered to 55% of the limit of quantitation (LOQ) to render the top 6,000 expressed genes. Filtered genes were normalized to Q3 (3^rd^ quartile of all selected targets). Hierarchical clustering was performed as quality control. CD68 ROIs were compared across regions using a linear mixed model (LMM) with Benjamini-Hochberg (BH) correction and a random effect for the region. For RNA sequencing pathway analysis, Wilcoxon rank sum test was used. In all cases, statistical significance was considered at p *≤* 0.05.

## Results

### Spatial discrimination of activated macrophages during SP-C^I73T^-induced injury

**Figure 1:**
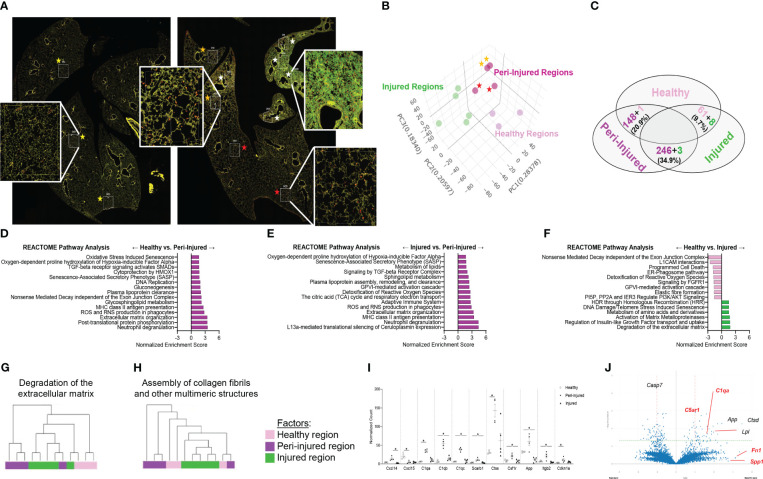
Spatial analysis of CD68^+^ macrophages in SP-C^I73T^ induced injury. Paraffin-embedded sections from controls or 14 days post SP-C^I73T^ injury were stained with fluorescent antibodies against CD45, CD68, and DNA. **(A)** Twelve alveolar regions were selected for CD68^+^ macrophage RNA sequencing (cells labeled in red). Regions from an untreated mouse were used as controls (three technical replicates; yellow asterisks). Injured/remodeled (five technical replicates; white asterisks) and peri-injured (two proximal technical replicates, orange asterisks; two distal technical replicates; red asterisks) region selection was based on histopathological assessment of inflammation and loss of alveolar architecture structure. Insets show representative regions. **(B)** Three dimensional PCA plot shows the clustering of samples based on sampling regions. Orange asterisks indicate two proximal technical replicates; red asterisks indicate two distal technical replicates. **(C)** Venn diagram of Reactome-based pathway analysis shows differentially regulated pathways between each pairing. **(D–F)** Bar graphs representing normalized enrichment scores for selected pathways. **(G-H)** Hierarchical clustering of ‘Degradation of the extracellular matrix’ and ‘Assembly of collagen fibrils and other multimeric structures’ signaling pathways in healthy (dark purple), peri-injured (pink), and injured (green) regions of the lung 14 days after SP-C^I73T^ induced injury. **(I)** Box plots of selected genes associated with macrophage activation (chemokine, metalloproteinases, complement cascade). The Y-axis represents normalized counts. A p-value ≤0.05 was considered significant using Linear Mixed Model. **(J)** Volcano plot comparing gene expression between peri-injured and injured macrophages. Fold changes are represented on log2 scale. Significance is shown as -log10(pvalue) using linear mixed model. In red are representative complement and fibrosis-associated genes.

To spatially define the phenotype of macrophages involved in fibrotic lung injury, fluorescent antibodies recognizing DNA (green), CD45 (yellow) and CD68 (red) were used to perform bulk sequencing in the injured/remodeled or peri-injured alveolar regions of the lung 14 days post SP-C^I73T^ injury. A control (non-induced) lung was used to define baseline activation. Identification of injured regions was based on histopathological evaluation of all five lobes in the tissue sections. Four peri-injured areas were selected: two in relative proximity to the injury (areas of interest 006 and 010) and two distal region (areas of interest 009 and 012) (**Figure 1A**). Cell-type deconvolution analysis was used to resolve monocyte/macrophage specific expression signature from the dataset (52) (**Supplementary Table 1**). Principal component analysis identified clustering of transcriptomes based on sampling annotation, with separation in the peri-injured regions based on proximity to injury (**Figure 1B**; **Supplementary Table 1**). Global pathway map showed extensive expression of genes involved in metabolism (including RNA metabolism), signal transduction, transcriptional regulation, immune system function and cell cycle (**Supplementary Figure 1A**). Reactome-based analysis revealed 20.9% (148/712 pathways) and 34.9% (249/712 pathways) of pathways as differentially expressed when comparing peri-injury vs. healthy macrophages and peri-injury vs. injured cells, respectively. Notably, macrophages isolated form injured regions did not produce a particularly strong signaling signature and demonstrated a considerable degree of similarity to healthy macrophages (≈90% of pathways) (**Figure 1C**; **Supplementary Figures 1B–D**). Peri-injured macrophages were enriched in pathways associated with neutrophil degranulation, ROS/RNS production and release, activation in oxidative stress-induced senescence, pro-remodeling functions (TGF-beta and GPVI cascade), and metabolic alterations (citric acid cycle, gluconeogenesis, lipoprotein assembly/remodeling/clearance) compared to healthy- and injured-derived CD68^+^ macrophages (**Figures 1D, E**; **Supplementary Table 1**). When compared to controls, injured region macrophages were defective in programmed cell death signaling, ROS detoxification, and elastic fiber formation, but displayed enhanced DNA damage-induced senescence, degradation of extracellular matrix (ECM), and metalloproteinase function (**Figure 1F**). Hierarchical gene expression analysis of pathways related to degradation of ECM (**Figure 1G**), assembly of collagen fibrils (**Figure 1H**), complement system, adaptive immunity, and L13a-mediated ceruloplasmin expression all showed robust clustering based on region of origin (**Supplementary Figures 1B–D**, **Supplementary Table 1**).

Among the top 6,000 genes expressed genes in the dataset, genes associated with complement responses (*C1qa, C1qb, C1qb*), myeloid cell recruitment (*Cxcl14, Cxcl15*), inflammation (*Csf1r, Ctss, App*) and metalloproteinases *(Mmp13, Mmp14, Mmp15*) were most abundant in peri-injured macrophages (**Figure 1I**). Despite increases in normalized counts for fibrogenic genes and significant enrichments in the associated pathways, expression of the individual genes did not meet significance (*Fn1, Spp1*) (**Figure 1J**; **Supplementary Figures 1E–G**).

### Temporal heterogeneity of resident alveolar macrophages during SP-C^I73T^-induced injury

**Figure 2:**
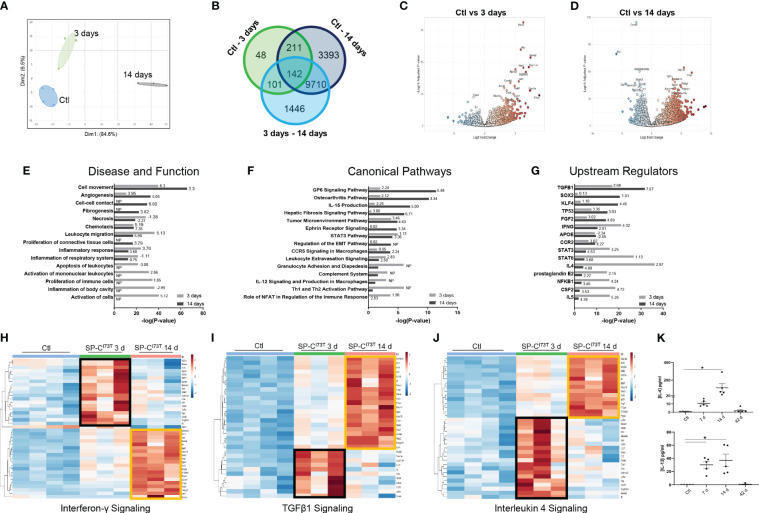
Transcriptional analysis of flow cytometry sorted alveolar macrophages following SP-C^I73T^ induced injury. Bulk RNA sequencing of flow cytometry sorted CD11b^-^SigF^+^CD11c^+^CD64^+^ resident lung macrophages from control (N=4; Ctl, oil treated SP-C^I73T^ mice) or tamoxifen-treated SP-C^I73T^ mice at 3 d and 14 d (N=3 for each condition). **(A)** Principal Component Analysis (PCA) plot showing transcriptome variance in Ctl (blue), 3 days (black), and 14 days post SP-C^I73T^ induced injury. **(B)** Venn diagram breaking down significantly expressed genes among groups. **(C, D)** Volcano plots comparing fold change expression between Ctl vs. 3 days and Ctl vs. 14 days. **(E-G)** Ingenuity Pathway Analysis (IPA) of enriched Diseases and Functions, Canonical Pathways, and Upstream Regulators in lung macrophages 3- and 14-days after injury relative to Ctl. Bars represent enrichment [−log(pvalue)] 3 days (gray) and 14 days (black) after injury. Z-scores indicate predicted activation and inhibition respectively (N.P. not predictable). **(H-J)** Heat maps depicting significantly altered genes associated with Interferon-γ, TGFβ1, and Interleukin-4 signaling 3- and 14-days after injury relative to Ctl; criteria for significance was a 5% false discovery rate. Note that orange and black boxes highlight signatures specific to a given time point. **(K)** ELISA for IL-4 and IL-13 from SP-C^I73T^ BAL fluid collected from controls, 7 days, 14 days, or 42 days post injury. Dot plots with Mean + SE are shown. *p < 0.05 versus control group using One Way ANOVA followed by Tukey *post-hoc* test.

To better understand macrophage behavior in the events leading up to end-stage fibrosis, we sought to examine transcriptional changes in resident macrophages using a model of fibrogenic injury triggered by mutant SP-C^I73T^. For these studies, we leveraged bulk RNA sequencing of flow cytometry sorted CD11b^-^SigF^+^CD11c^+^CD64^+^ resident mature macrophages isolated from naïve (controls) and inflamed lungs. Exclusion gate ensured no contamination from Ly6G^+^ neutrophils, B220^+^ B cells, and CD3^+^ lymphocytes. Ingenuity Pathway Analysis highlighted distinct gene and signaling signatures in lung macrophages during inflammatory initiation (3 days post mutant induction) and early remodeling (14 days). Principal component analysis highlighted transcriptional variance across the study groups (**Figure 2A**). Differential gene expression analysis revealed a relatively small set (48 genes) between controls and 3-day injury, while these responses were more pronounced between controls vs. 14-day post induction (3393) or 3-day vs. 14-day comparison (1446) (**Figure 2B**). Volcano plots showed increases in genes linked to innate immunity (the hematopoietic transcription factor, *Gata2;* histidine decarboxylase, *Hdc; interferon induced transmembrane protein 1, Ifitm1;* colony stimulating factor 1*, Csf1;* chemokine ligand*, Ccl17;* immunoglobulin epsilon receptor, *Ms4a2) and* metabolism *(*cholesterol side-chain cleavage enzyme*, Cyp11a1;* ATPase Na+/K+ transporting subunit alpha 3, *Atp1a3;* adenylate cyclase 6, *adcy6;* peptidyl arginine deiminase 2*, Padi2;* and myristoylated alanine rich protein kinase C substrate, *Marcks)* 3 days post injury (**Figure 2C**). By comparison, macrophage expression profile was bidirectional at 14 days, and demonstrated more sizable changes (as represented by adjusted p-values and log fold changes). Immunity, cell cycle, and metabolism genes were among the most significantly altered genes (complement C1q C-chain, *C1qc*; peptidoglycan recognition protein 1, *Pglyrp1*; ADAM metallopeptidase domain 19, *Adam19*; secreted protein acidic and cysteine rich, *Sparc*; apolipoprotein E, *Apoe*; insulin like growth factor 1 receptor, *Igf1r*; toll-like receptor 7, *Tlr7*; cyclin D2, *Ccnd2* (**Figure 2D**). Ingenuity Pathways Analysis predicted signaling related to activation, proliferation, and apoptosis of leukocyte predominantly at 3 days, while distinct cell movement and chemotaxis pathways, proliferation, angiogenesis and fibrogenesis were projected to be induced 14 days post-induction (**Figure 2E**; **Supplementary Table 2**). Specific pathways linked to NFAT dependent regulation of immune responses (directionality/z-score 1.90), Th1 and Th2 activation (no predicted z-score), granulocyte adhesion and diapedesis (no predicted z-score), and STAT3 signaling (z-score 1.13) were noted 3 days post injury. Comparatively, glycoprotein 6 (z-score 5.48), IL-15 production (z-score 5.00), fibrosis (z-score 6.71) and epithelial-mesenchymal transition signaling (no z-score), and osteoarthritis (z-score 3.34) were identified in macrophages at 14 days (**Figure 2F**). Notably, IPA’s Upstream Regulators analysis predicted activation of pro-inflammatory signals at 3 days (NFKB1, IL5, IFNG, prostaglandin E2, STAT3 and STAT6), and conventional fibrogenic pathways (TGFB1, TP53, FGF2, and SOX2) 14 days (**Figure 2G**). Reactome-based breakdown of top differentially expressed genes from the Interferon-γ pathway identified distinct gene-sets expressed during inflammatory initiation (*Ifi203, Ifi206, Ifi209, Ifi213*, *Stat4)* and 14 days post injury (*S100a8, Arg1, Csf1, Alox15, Retnla, Il4)* (**Figure 2H**; **Supplementary Table 2**). A comparable dual response was observed in the TGF-β1 (3 days: *Ms4a2, Ctsk*, C*lec2i* and Cd55; 14 days: *Il1rl1*, A*rg1, Alox15, F13a1, Itgam*, P*tgs2, C1qc*, and A*nxa8*) and IL-4 signaling pathways (3 days: *Ms4a1, Cxcl5, Il6, Stat4*; 14 days: *S100a8, Agf2r, Csf1, Retnla, Il4)* (**Figures 2I, J**). ELISA-based validation of this established pro-fibrotic pathway confirmed increases in IL-4 and IL-13 expression in the bronchoalveolar lavage fluid 7-14 days post SP-C^I73T^ injury (**Figure 2K**). Notably, IL-15 and Glycoprotein-6 signaling, as well as angiogenesis displayed time restricted enrichments (14 days post-induction) (**Supplementary Figures 2A, B**, and not shown).

### Single-cell RNA sequencing reveals activation of distinct monocyte/macrophage clusters responding to SP-C^I73T^ induced injury

**Figure 3:**
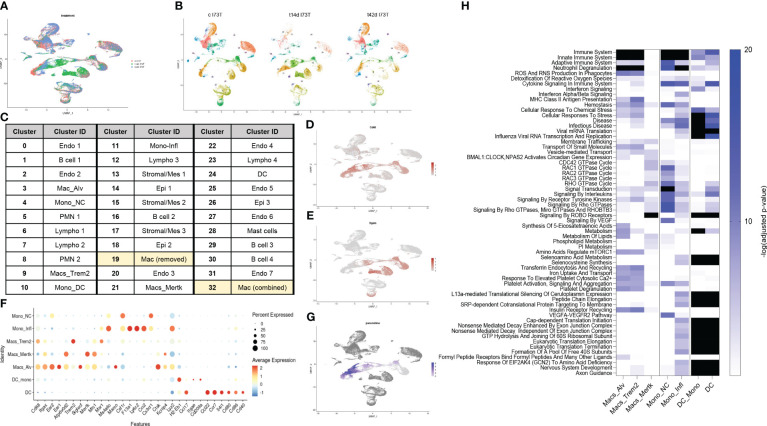
Single-cell RNA sequencing of SP-C^I73T^ induced injury and fibrosis. Single-cell RNA sequencing analysis was performed on collagenase digested single cell suspensions from controls (Ctl, oil treated SP-C^I73T^ mice), 14 days or 42 days post SP-C^I73T^ induction. **(A)** UMAP analysis overlaying cells from controls (cI73T, pink), 14 days (t14d I73T, green), and 42 days (t42d I73T, blue). **(B)** Split identify UMAP depiction of all cellular clusters identified in controls (cI73T), 14 days (t14d I73T), and 42 days (t42d I73T) at 0.4 resolution. **(C)** Cluster breakdown identifying 7 endothelial, 3 mesenchymal, 3 epithelial, 2 polymorphonuclear, mast cells, 4 B cell, 4 lymphocytic, and 9 mononuclear myeloid clusters. Note that mononuclear myeloid clusters were renamed based on single R and manual annotation: cluster 3 = Mac_Alv; cluster 4 = Mono_non classical/NC; cluster 9 = Mac_Trem2; cluster 10 = Mono_DC; cluster 11 = Mono_inflammatory/infl; cluster 19 = was removed from further analysis as it was identified solely in one of the controls; cluster 21 = Mac_MerTK; cluster 24 = DC; cluster 32 = was combined with cluster 3/Mac_Alv since pseudobulk analysis revealed analogous gene expression and pathway activation. **(D, E)** UMAP analysis for *Cd68* and *Itgam/Cd11b* defines distribution and cellular expression in the SP-C^I73T^ lung. **(F)** Bubble plot of selected genes associated with macrophage maturation (*Trem2, Itgax, Ear1, Ear2, Atp6v0d2, Siglecf, Mertk*), recruitment and activation (*F13a1, Ly6c2, Ccr2, Cx3cr1, Ctsk, Lyz2, H2-eb1, CcL22, Ccl17, Cd80, Cd86)* in all annotated monocytes/macrophages/dendritic cell clusters (Mo/Mac/DCs). **(G)** Pseudotime UMAP analysis. Note color intensity defining cellular maturation among monocyte/macrophage clusters. **(H)** Enrichr-based Reactome pathway analysis for the top-15 predicted pathways in the 7 resulting monocyte/macrophage/DC clusters. Single gradient color heatmap shows logarithmic adjusted p-values. Values above the arbitrary threshold (-log of adjusted p-value) of 20 were color-coated in black.

We then employed single-cell RNA sequencing to overcome the constraints (and therefore bias) of antibody-based analysis of macrophages involved in SP-C^I73T^ induced injury. A 59,440-cell dataset including healthy controls (29,213 cells), and two injury times representing peak inflammation (14 days - 14,266 cells) and established fibrosis (42 days - 15,961 cells) were studied. Population clustering using SCTransformation with the glmGamPoi method at a resolution of 0.4 yielded 33 clusters (**Supplementary Figure 3A**). Partition of the clusters based on origin/identity (Ctl, 14 days, 42 days) highlighted shifts in endothelial cells (cluster 25), eosinophils (cluster 8), and macrophages (cluster 9) composition after SP-C^I73T^ injury (**Figures 3A, B**). A combination of SingleR, manual annotation, and the top three non-redundant genes from each populations was used for identification of epithelial (3), endothelial (6), mesenchymal/stromal (3), megakaryocytes, granulocytes (3), B cells (4), lymphocytes (4), and mononuclear myeloid populations (9) (**Figure 3C**; **Supplementary Figures 3B–I**). Macrophage Cluster 19 (identified in only one of the eleven specimens) and Cluster 32 (of low abundance and merged with Cluster 3 after pseudobulk analysis determined high degree of transcriptional overlap).

Uniform Manifold Approximation and Projection (UMAP) analysis for the pan-macrophage gene *Cd68* and the mobilization marker *Itgam/Cd11b* were utilized to locate resident and recruited macrophages, monocyte-derived, and by exclusion monocytes and dendritic cells (**Figures 3D, E**). A curated gene set (mobility, maturity, and activation markers) combined with pseudotime analysis was used to clearly differentiate mononuclear myeloid clusters (**Supplementary Table 3**). Cluster 3 was annotated as alveolar macrophages since it expressed a combination of *Cd68, Itgax, Ear1*, *Ear2, Siglecf*, and moderate levels of *Mertk* (**Figure 3F**). Reconstructed trajectory analysis also predicted terminal differentiation within the alveolar macrophage cluster (**Figure 3G**). Cluster 9, identified as interstitial macrophages (*Cd68*, M*sr1, and C1qa*), was labeled based on its specific expression of *Trem2* (**Figure 3F** and not shown). Cluster 21 (macs_mertk) was identified as a mature subset based on pseudotime analysis and distinctive expression of *Mertk* alongside *Itgax*, *Mrc1*, *Atp6v0d2*, *Kcnip4.* This cluster was found solely in controls and 42 days post injury (**Figures 3F, G**). Two monocyte-like subsets were annotated as non-classical/NC (cluster 4 - *Csf1r, Cx3cr1*) and classical/inflammatory (cluster 11 - C*cr2, Ly6c2, Lyz2, Ms4a6c, F13a1*) (**Figure 3F**; **Supplementary Table 3**). Cluster 10 was annotated as monocyte-derived DCs based on a mixed myeloid and lymphoid signature (*Ccr2*, *Itgae/Cd103*, *Cd209/Dc-sign)* and major histocompatibility complex genes. A cluster of lymphoid-derived DCs (cluster 24) lacking all classical myeloid features and expressing *Cd80, Cd86, Cd40, Il4i, Ccr7, Ccl17*, and *Ccl22* was also annotated (**Figure 3F**). Split identity analysis of these curated gene sets also highlighted time related changes in the expression of established macrophage maturity and identity genes (e.g. *Atp6v0d2, Itgax*, and *Cd68* expression increases, downregulation of *Siglecf, Ear1*, and *Ear2*) (**Supplementary Figure 4A**). Similar trends were observed for neutrophils (PMN 1 lost *Ptgs2* and *Tgm2* in favor of *Retnlg* and *Lcn2), e*osinophils (PMN 2 - *Siglecf, Itgam, Cxcr2, Cd33, Csf3r, Tgfbr1*), epithelial cells (Epi1 - *Cldn18, Ager, Hopx*, *Krt8, Krt19, Sftpb*), mesenchymal/stromal cells (Mes 2 - *Tgfbr3, Ccn1, Pdgfra, Npnt*, *Loxl1, Ecm1, Fgf2*), and lymphatic endothelial cells (Endo 6 - *Nrgn, Itga2b, Gp1bb* increased 14 days post injury). By comparison, B and T cells showed limited transcriptional fluctuation over the 42-day injury (**Supplementary Figures 4B–G**).

Pseudobulk differential expression analysis of *Trem2* ^+^ macrophages surveyed gene expression and patterns during injury. Approximately 700 genes demonstrated a transient drop in abundance at 14 days (expression pattern group-1), while the abundance of 621 genes was significantly increased following SP-C^I73T^ induction (expression pattern group-2) (**Supplementary Figure 5**). Smaller gene sets were shown to transiently increase at 14 days (122 genes, expression pattern group-3) or steadily decrease after SP-C induction (61 genes, expression pattern group-4) (**Supplementary Figure 5**). Notably, Reactome-based analysis predicted no significant pathway to be altered for genes annotated in expression pattern-1, while cytokine and interleukin signaling, immunity, antigen presentation, and lipid and carbohydrate metabolism were among significant pathways for expression pattern-2 genes. By comparison, transcript abundance in inflammatory monocytes followed two expression patterns: transient decrease (237 genes) or increase (158) at 14 days post injury. The latter predicted engagement of pathways related to complement cascade, extracellular matrix organization and immune responses (**Supplementary Table 3**).

Reactome-based analysis of the top-15 most significantly regulated pathways revealed shared inflammatory signature (‘immune system’, ‘neutrophil degranulation’, ‘ROS and RNA production in phagocytes’) between alveolar macrophages and *Trem2* ^+^ interstitial macrophages, though at considerably different adjusted *p*-values (Alv macs *p*-value <10^-25^ vs. *Trem2^+^ p*-value <10^-103^) (**Figure 3H**; **Supplementary Table 3**). By comparison, *Mertk^+^* cells were not predicted to engage in innate or adaptive immunity or cytokine mediated signaling, but generated a strong GTPase signature (CDC42, RAC, RHO, and ROBO/SLIT). Examination of monocyte-like clusters highlighted analogous ‘cytokine signaling in immune system’, ‘innate immunity’, and ‘neutrophil degranulation’ engagement. *Cx3cr1* ^+^ non-classical cells excelled in ‘VEGF signaling’, ‘signaling by tyrosine kinases’, and ‘Rho GTPase signaling’, while *Ccr2^+^Ly6c2^+^* classical/inflammatory monocytes produced a signature related to RNA synthesis and translation and pathways involved in ‘cellular response to stress’. Lastly, monocyte-derived and lymphoid DCs produced comparable activation profile (infectious disease response, translational elongation and termination, and ribosomal homeostasis) (**Figure 3H**).

### Macrophages from human IPF display a comparable phenotype as murine SP-C^I73T^ injury.

**Figure 4:**
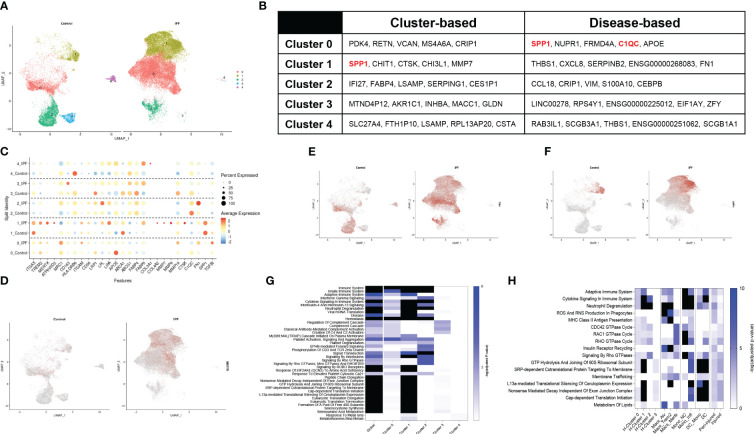
Single-cell RNA sequencing of annotated macrophages from human IPF. Macrophages from a published IPF dataset (GSE136831) were re-mined. **(A)** Split identity UMAP analysis of clusters annotated at 0.1 resolution using the Seurat analysis package. **(B)** Cluster-based and disease-based top-5 non-redundant genes from annotated clusters. **(C)** Split identity UMAP shows expression distribution for *TREM2* in control and IPF lungs. **(D)** Split identity bubble plot of selected genes associated with macrophage maturation (*TREM2, MERTK, ATP6V0D2, ITGAX*), recruitment and activation (*ITGAM, CD163, HLA-DRB6, CTSK, COL3A1, COL4A2, MMP7, MMP9, FN1, SPP1, TGFBI*), and lipid homeostasis (*APOE, CD36, LRP1, LPL, LIPA, ABCA1, ABCG1, FABP5, PPARG*). **(E)** Split identity UMAP showing expression distribution for *MERTK* in control and IPF lungs. **(F)** Split identity UMAP showing expression distribution for *FN1* in control and IPF lungs. **(G)** Split identity UMAP showing expression distribution for *SPP1* in control and IPF lungs. **(H)** Enrichr-based Reactome pathway analysis for the top-15 predicted pathways between controls and IPF samples. Single gradient color heatmap shows logarithmic adjusted p-values. Values above the arbitrary threshold (-log of adjusted p-value) of 6 were color-coated in black. **(I)** Enrichr-based Reactome pathway analysis comparing significant pathways in mononuclear myeloid clusters from the human IPF dataset, SP-C^I73T^ spatial transcriptomics, and SP-C^I73T^ single-cell sequencing datasets. Single gradient color heatmap shows logarithmic adjusted p-values. Values above the arbitrary threshold (-log of adjusted p-value) of 10 were color-coated in black.

A recent dataset published by Adams and colleagues provided in depth assessment of epithelial, endothelial, mesenchymal, and immune populations in healthy and IPF tissue explants (40). The mononuclear myeloid compartment from this set (44,226 cells; 12,514 from controls and 31,712 IPF, GSE136831) was re-examined to define similarities between clinical and experimental fibrosis. To avoid overfitting the data, the lowest resolution was utilized (0.1), ultimately generating 5 major subsets (**Supplementary Figure 6A**). UMAP analysis identified clusters 3 and 4 solely in the controls, with an expansion in clusters 0 and 1 was noted in IPF (**Figure 4A**). Cluster-based analysis of top-5 non-redundant genes revealed differential expression for *SPP1, CTSK, MMP7* (cluster 1) and *FABP4* (cluster *2*) (**Figure 4B**; **Supplementary Table 4**). Disease-based analysis highlighted notable changes in cluster 0 (*SPP1, C1QC, APOE*) and cluster 1 (*THBS1, FN1*), consistent with our murine dataset, including, (**Figures 4B, C**, **Supplementary Table 5**). A curated set of 25 identity and lipid associated genes, used to annotate this clusters, identified cluster 0 as an interstitial macrophage population (widespread expression of *ITGAX*, *TREM2*, *MRC1, ITGAM*, *APOE)* and Cluster 1 as alveolar macrophages (*ITGAX/CD11C, ITGAM*, *ABCA1*). Notably, cluster 1 also displayed a signature characteristic of a potentially pro-fibrotic monocyte-derived population in IPF lungs (*TREM2*, *MERTK*, *ATP6V0D2, CTSK*, *COL4A2, MMP7, MMP9)* paired with a shift in metabolic function (*FABP5, LPL* and *LIPA* increases). Clusters 2, 3 and 4 did not exhibit distinguishing signatures besides overexpression of major histocompatibility complex genes (*HLA-DRB6*) (**Figure 4C**; **Supplementary Figure 6B**). UMAP analysis of the distribution of *MERTK* and fibronectin1/*FN1* demonstrated widespread presence in cluster 0 and 1 of the IPF lung, while ostepontin1/*SPP1* was restricted to cluster 1 (**Figures 4D–F**). Tumor growth factor β1/*TGFβ1* was expressed in all cells regardless of disease state (**Supplementary Figures 6C, D**). Enrichr-based pathway analysis using the Reactome database highlighted IPF induced changes (annotated as “global”) in all facets of immune cell behavior, including “Immunity”, “Cellular Responses to Stress”, “Cytokine Signaling in Immune System”, complement activation, and GTPase signaling, and control of transcription and translation. Clusters 0, 1, 2 were responsible for the majority of these signals (**Figure 4G**; **Supplementary Table 4**). Direct comparison of Reactome pathways from the spatial analysis and both the murine and human single-cell sequencing datasets highlighted similarities between cluster 1 annotated in the human IPF dataset and inflammatory monocytes from the SP-C^I73T^ mouse model. No distinctive similarities were noted with respect to CD68^+^ macrophages sampled from peri-injured and injured regions of the lung during our spatial analysis (**Figure 4H**).

### Osteopontin1 and fibronectin1 intercellular communication during SP-C^I73T^ induced injury

CellChat software was utilized to predict communication among cellular clusters during SP-C^I73T^ induced fibrotic injury. Initial analysis grouped all 33 clusters into 7 macro-groups (epithelial, endothelial, stromal, B cells, granulocytes, lymphocytes, and Mo/Mac/DCs) (**Supplementary Figure 7A**) (51, 53). CellChat based aggregation of ligand: receptor expression estimated increases in differential number and strength of interaction between mesenchymal-epithelial and mesenchymal-Mo/Mac/DCs and within epithelial clusters 14 day post-injury, relative to controls. Within the Mo/Mac/DC macro-groups the communication was estimated to increase in strength (**Supplementary Figure 7B**). Interrogation of the inter-cluster network 42 days post-induction showed increased interactions in stromal and Mo/Mac/DC macro clusters relative to controls and 14 days (**Supplementary Figures 7C, D**), with incoming and outcoming signals from the Mo/Mac/DC macro-cluster associated with SPP1 and FN1 pathway (**Supplementary Figures 7E–G**, **Supplementary Tables 6**–**9**).

**Figure 5:**
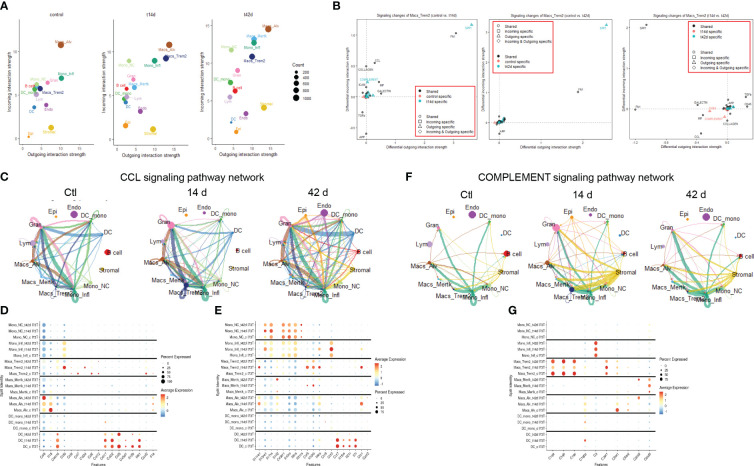
Cell-cell communication analysis in SP-C^I73T^ induced injury and fibrosis. CellChat software was used to estimate cell-cell communication in controls (Ctl, oil treated SP-C^I73T^ mice), 14 days or 42 days post SP-C^I73T^ induction. **(A)** Ligand:receptor expression analysis examining incoming (y-axis) and outgoing (x-axis) signals in controls, 14 days or 42 days post injury. Note that individual endothelial, mesenchymal, epithelial, granulocyte, B cell, and lymphocyte clusters were combined into “macro-clusters”, while mononuclear myeloid cells remained split. The size of the circles is representative of population size. **(B)** Prediction of signaling changes in *Trem2* ^+^ macrophages between control and 14-day, control and 42-day, and 14-d and 42 d. Plot legend describes directionality (circle - shared by both groups, square - incoming in a specific group, triangle - outgoing in a specific group, diamond - incoming and outgoing in a specific group) and signal specificity (black - shared by both groups, orange - control specific, cyan - injury/tamoxifen specific). **(C)** Connectome ring plots for chemokine ligand/CCL signaling pathway network predict directionality and communication strength among clusters. **(D, E)** Split identity bubble plot for chemokine/cytokine/interleukin ligands and receptors among mononuclear myeloid clusters. Note that the size of the bubble indicates the relative abundance of the population expressing target gene. Color coating indicates average expression. **(F)** Connectome ring plots for Complement signaling network predicting directionality and strength of communication among clusters. **(G)** Split identity bubble plot for complement associated genes among mononuclear myeloid clusters. Note that the size of the bubble indicates the relative abundance of the population expressing target gene. Color coating indicates average expression.

To pinpoint the exact cellular origin of these signals, CellChat analysis was carried out after split of the Mo/Mac/DC macro-cluster. This analysis revealed alveolar macrophages as a dynamic cluster at baseline (both incoming/receptor-based and outgoing/ligand-based interactions), while *Trem2* ^+^ macrophages produced high volume of incoming/outgoing signals 14 days post injury and *Mertk* ^+^ cells became active at 42 days. Classical/inflammatory monocytes maintained comparable network profile throughout the 42-day time course, with non-classical monocytes effectively reducing outgoing signals after SP-C^I73T^ injury (**Figure 5A**; **Supplementary Figures 8A–C**). Split identity pathway analysis across the 42-day time course demonstrated the origin of the osteopontin1/SPP1, fibronectin1/FN1, chemokine ligand/CCL, laminin, semaphorin3/SEMA3, galectin, complement, and collagen signaling to be driven by *Trem2* ^+^ macrophages at 14 days (**Figure 5B**). By 42 days, the predicted interaction strength was comparable across the monocyte and macrophage clusters (**Supplementary Figures 8D–F**). Connectome ring plots were used to capture the changes and directionality of these interactions during injury and fibrosis. Analysis of CCL signaling predicted baseline crosstalk among all annotated mononuclear myeloid cells and granulocytes. While these responses were unaffected 14 days post SP-C^I73T^ injury, by 42 days there was a global activation of this signaling network (**Figure 5C**). Differential expression of chemokines/cytokines/interleukins and their receptors highlighted distinct signatures in alveolar macrophages (*Ccl6*, *Il18, Cxcl2*, and *Il1a)*, *Trem2* ^+^ macrophages (*Ccl12, Ccl2, Ccl24, Ccl9*, *Cxcl16, Ccr5, Il10rb, Il11ra1), while Mertk* ^+^ macrophages and monocyte-derived DCs were relatively quiescent. Classical and inflammatory monocytes presented a receptor dominant repertoire (*Cx3cr1, Il10ra, Il17ra, Il6ra –* inflammatory monocytes exclusively expressed *Ccr2)* (**Figures 5D, E**). CellChat-based network analysis for complement signaling predicted outgoing communication from classical monocytes to other myeloid clusters in all conditions, with a transient activation originating from stromal cells 14 days post injury (**Figure 5F**). Differential expression revealed distinct signatures in *trem2* ^+^ macrophages (*C1qa, C1qb, C1qc, C3ar1*) and stromal cells (stromal 2 - *C1qtnf7, C1ra, C2, C3, C4b, C7*), with granulocytes also expressing *C3ar1*, *C5ar1*, and *C5ar2* (**Figure 5G**).

### Trem2^+^ macrophages coordinate pro-fibrotic communication during SP-C^I73T^ induced injury and fibrosis

**Figure 6:**
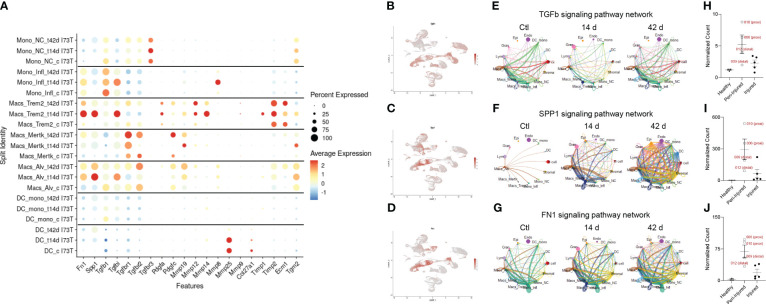
Pro-fibrotic cell-cell communication in SP-C^I73T^ induced injury and fibrosis. CellChat-based analysis of fibrogenic signaling from controls (Ctl, oil treated SP-C^I73T^ mice), 14 days or 42 days post SP-C^I73T^ induction. **(A)** Split identity bubble plot for fibrosis-associated genes among mononuclear myeloid clusters. Note that the size of the bubble indicates the relative abundance of the population expressing target gene. Color coating indicates average expression. **(B-D)** UMAP analysis for *tgfb1, spp1, fn1.* **(E-G)** Connectome ring plots for TGFb, osteopontin/SPP1 fibronectin/FN1 signaling pathway network predicting directionality and communication strength among clusters. Note that only Mo/Mac/DC clusters are split. **(H-J)** Box plots for *Tgfb1, Spp1, Fn1*. CD68^+^ macrophages isolated from healthy regions are shown in dark purple, peri-injured macrophages in pink, and injured macrophages are shown in green. The Y-axis represents normalized counts.

**Figure 7:**
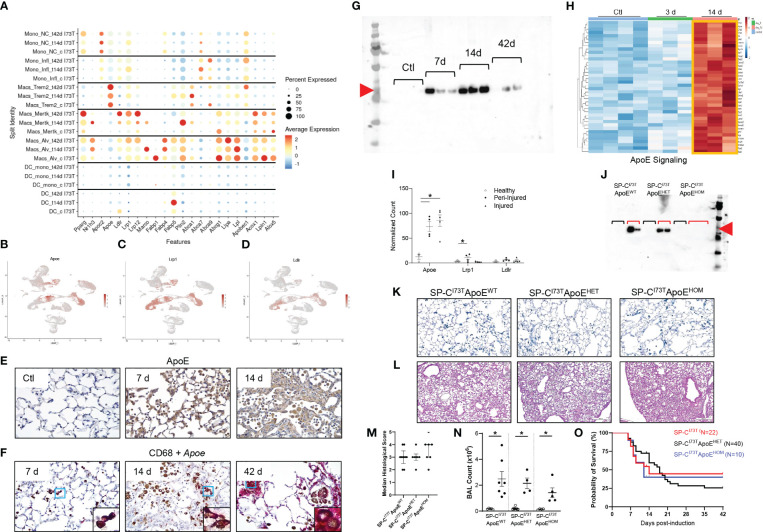
Lipid signature in SP-C^I73T^ induced injury and fibrosis. Lipid-associated gene expression was assessed in controls (Ctl, oil treated SP-C^I73T^ mice), 14 days or 42 days post SP-C^I73T^ induction. **(A)** Split identity bubble plot for lipid-associated genes among mononuclear myeloid clusters. Note that the size of the bubble indicates the relative abundance of the population expressing target gene. Color coating indicates average expression. **(B-D)** UMAP analysis for *Apoe, Lrp1, Ldlr.* **(E)** Immunohistochemical analysis for ApoE in controls, 14 days or 42 days post SP-C^I73T^ induced injury. A representative image is shown. **(F)** Combination of immunohistochemistry (CD68, brown) and *in situ* hybridization (*Apoe*, magenta). Protein expression was visualized using a DAB Vectastain kit. Original magnification, 400x. Insets, 1000x. **(G)** ApoE western blot analysis of BAL fluid from SP-C^I73T^control (Ctl), 7 days, 14 days, and 42 days post induction (N=3). Red arrowhead indicates molecular weight band for ApoE (34 KDa). **(H)** Heat maps of significantly altered genes associated with ApoE signaling in flow cytometry sorted CD11b^-^SigF^+^CD11c^+^CD64^+^ resident lung macrophages 3- and 14-days after injury. The criteria for significance was a 5% false discovery rate. Note that the orange box highlights signatures specific to a given time point. **(I)** Box plots for *Apoe, Lrp1, Ldlr*. The Y-axis represents normalized counts. A p-value ≤0.05 was considered significant using Linear Mixed Model. **(J)** ApoE western blot analysis of BAL fluid from control and 14 days SP-C^I73T^ApoE^WT^ (controls N=2; 14 days N=2), SP-C^I73T^ApoE^HET^ (controls N=2; 14 days N=2), and SP-C^I73T^ApoE^HOM^ (controls N=2; 14 days N=3). Red arrowhead indicates molecular weight band for ApoE (34 KDa). Black brackets represent ApoE^WT^, Apoe^HET^, ApoE^HOM^ BAL fluid from control SP-C^I73T^ mice. Red brackets represent ApoE^WT^, Apoe^HET^, ApoE^HOM^ BAL fluid 14 days post SP-C^I73T^ induced injury. **(K)** Duplex *in situ* hybridization analysis for *Tgfb1* (pink) *Spp1* (blue) in SP-C^I73T^ApoE^WT^, SP-C^I73T^ApoE^HET^, and SP-C^I73T^ApoE^HOM^ lungs 14 days post injury. Representative images are shown. **(L-M)** H&E staining and histological scoring of SP-C^I73T^ApoE^WT^, SP-C^I73T^ApoE^HET^, and SP-C^I73T^ApoE^HOM^ lungs 14 days post injury. Pathological scoring included counting of foci of injury, extent of inflammation, edema, hemorrhage, and alveolar architecture remodeling. Representative images are shown. **(N)** Bronchoalveolar lavage fluid (BAL) cell counts of SP-C^I73T^ApoE^WT^, SP-C^I73T^ApoE^HET^, and SP-C^I73T^ApoE^HOM^ samples (controls and 14 days post injury). Data are presented as mean ± SEM (n = 4-9 mice/group), analyzed using two-way ANOVA. A p<0.05 (*) was considered significant. Lines mark significant groups. **(O)** Kaplan–Meier survival curve of SP-C^I73T^ApoE^WT^, SP-C^I73T^ApoE^HET^, and SP-C^I73T^ApoE^HOM^ lungs 14 days post injury. Analysis includes mice found dead or displaying ≥20% body weight from study initiation. Log-Sum (Mantel-Cox) Rank test was used.

We then query the dataset for distinctive extracellular matrix reorganization gene signatures. Unsurprisingly, mesenchymal cells produced signals from collagen genes, metalloproteinases, laminins, and platelet-derived growth factor receptor alpha and beta (**Supplementary Figure 9**). Expression of *Timp1, Mmp12*, and *Mmp14 were restricted to Trem2* ^+^ cells, while *Spp1, Fn1*, and *Mmp19* were shared with alveolar macrophages (**Figure 6A**)*. Tgfb1* transcripts were maximal in inflammatory monocytes, though UMAP analysis of distribution suggested widespread expression in the lung (**Figures 6A, B**). Notably*, Spp1*, and *Fn1* expression was also found in the endothelial and mesenchymal compartment (**Figures 6C, D**). Signaling connectome ring for *Tgfb1* highlighted baseline signals emanating primarily from alveolar macrophages, non-classical monocytes, monocyte-derived DCs, and B cells. Despite transient drop 14 days post SP-C^I73T^ induced injury, the network returned to control levels by 42 days, with involvement of *Mertk* ^+^ macrophages (**Figure 6E**; **Supplementary Tables 6**–**9**). Osteopontin1 signaling network revealed alveolar macrophages as the sole driver in control lungs. SP-C^I73T^ induced injury produced increases in alveolar and *Trem2* ^+^ macrophages (14 days), and ultimately global expression in all lung cells (42 days) (**Figure 6F**; **Supplementary Tables 6**–**9**). Inflammatory monocytes and alveolar macrophages were shown to engage in fibronectin 1 signaling in the control and injured lung. Notably, *Trem2* ^+^ macrophages and stromal cells were predicted to partake in FN1 communication 14 days post-injury, while *Mertk^+^* macrophages were shown at 42 days (**Figure 7G**; **Supplementary Tables 6**–**9**). Although spatial examination did not detect significant differences among CD68^+^ macrophages collected from healthy, peri-injured, and injured regions of the lung 14 days post injury, we found that proximity to the remodeled tissue (areas of interest 006 and 010) resulted in higher expression of *Tgfb1*, *Spp1*, and *Fn1* (**Figures 6H–J**).

### Genetic ablation of ApoE does not impact SP-C^I73T^ induced injury

Curated analysis for genes involved in lipid handling and metabolism showed heterogeneous expression among mononuclear myeloid cells during SP-C^I73T^ induced injury and fibrosis. Monocytes exhibited a limited repertoire of lipid-associated genes. Alveolar macrophages displayed an extensive signature at baseline (*Fabp1, Plin2, Lipa, Lpl, Lpin1, Abcg1, Acox1*) which expanded after injury (*Nr1h3, Marco, Fabp4*). *Mertk* ^+^ macrophages developed distinct signatures at 14 days (*Nr1h3, Marco, Plin2, Abca1*) and 42 days (*Pparg Ldlr*, *Lrp1, Lrp12*, and *Acox1*), a time coordinated with their maximal abundance (**Figure 7A**). *Trem2^+^* gene expression was restricted to ATP binding cassette transporters (*Abca1, Abca9*) and exclusive *Apoe* expression. Analysis of the distribution of *Apoe* and two of its established receptors (*Lrp1, Ldlr*) confirmed predominant expression in mononuclear myeloid and mesenchymal clusters (**Figures 7B–D**) (54). Immunohistochemical analysis of ApoE showed increases in parenchymal and immune cells 7 days and 14 days post induction, in particular within injury foci (**Figure 7E**). *In situ* hybridization analysis validated the origin of *Apoe* in CD68^+^ macrophages up to 42 days after SP-C^I73T^ injury, while western blot analysis of BAL fluid also confirmed protein increases after SP-C^I73T^ induced injury (**Figures 7F, G**). Our bulk RNA sequencing predicted activation of the ApoE signaling pathway in SigF^+^CD11c^+^CD64^+^ alveolar macrophages, however its expression (or that of its receptors) was not spatially restricted (**Figures 7H, I**). To test the hypothesis that ApoE is directly implicated in Mo/Mac/DC function during pulmonary remodeling, mono- and bi-allelic deletion of ApoE (SP-C^I73T^ApoE^HET^ and ApoE^HOM^) was designed (**Figure 7J**). Neither ApoE hypofunctional or null mice impacted accumulation of *Spp1* ^+^ macrophages within areas of injury 14 days post SP-C^I73T^ induction (**Figure 7K**). Similarly, there was no improvements in pulmonary histopathological scoring, BAL cell counts, or mortality (**Figures 7L–O**).

## Discussion

Genetic mutations in key nodes of pulmonary epithelial function are intertwined with familial forms of pulmonary fibrosis (PF) and other interstitial lung diseases (55). Dysbiosis among parenchymal cells promote repeated cycles of injury, inflammation, epithelial-to-mesenchymal transition, and the proliferation of polyclonal fibroblast clusters, leading to spatially and temporally heterogenous tissue remodeling (56–58). In the current paradigm of PF, the role of immune cells remains ambiguous and primarily circumscribed to “acute inflammatory exacerbations”, sporadic events that severely worsen disease phenotype and accelerate patient mortality. This paradigm is supported by clinical and experimental evidence linking excess inflammatory monocyte mobilization and their retention as pro-fibrotic monocyte-derived macrophages to poor prognosis (13, 14, 20, 22). Our group has previously defined the involvement of mononuclear myeloid cells at all stages of inflammatory exacerbations triggered by a clinically relevant single point mutation in the alveolar epithelial cell specific gene encoding for the surfactant protein C (SP-C^I73T^) (59). Using this experimental platform and available human IPF datasets, this work aimed to expand the current understanding of PF and fill an important gap pertaining to macrophage phenotypic heterogeneity and aberrant cell-cell crosstalk in fibrotic disease. Specifically, we spatially characterized the transcriptional profile of CD68^+^ macrophages within and surrounding fibrotic foci of injury; defined incoming and outgoing pro-fibrotic communications among pulmonary cells; identified a dynamic lipid signature across all annotated macrophage and monocyte clusters, with a sole population producing *Apoe*; and showed that SP-C^I73T^ induced fibrosis is marginally impacted by genetic *Apoe* deletion.

Initial work was designed to spatially localize activated macrophages in the healthy, inflamed, and fibrosing lung. This approach aligns with a handful of recent reports that elegantly resolve epithelial, mesenchymal, and immune cell identity and abundance in clinical and experimental fibrosis (60, 61). Our studies go beyond the focus of these publications by placing emphasis on the distribution of macrophages from spatially diverse regions of the injured lung. CD68 represents a compromise allowing to sample both control/healthy and injured macrophages. Our gene expression and hierarchical clustering analysis demonstrate heterogenous transcriptional signatures among macrophages sampled from the fibrosing lung, thus suggesting a gradient of activation based on spatial localization. Pathway analysis also painted an unexpected picture characterized by transcriptionally quiescent CD68^+^ cells sampled from fully remodeled regions, contrasting a hyperactive phenotype emanating from peri-injured macrophages. Within this region of interest our findings also support the notion that macrophages activation is linked to their proximity to the injury. Findings that innate and adaptive immunity, matrix remodeling, senescence, and redox balance signals are predicted to be driven primarily by peri-injured macrophages (compared to all other sampled regions) adds depth to previous reports showing unequal expression of inflammatory proteins among immune cells found in healthy and fibrotic regions of the clinically diseased lung (62). Our results also support and complement evidence showing downregulation of inflammatory signaling (TNFα) in IPF immune infiltrates compared to healthy/unaffected regions (63). The comprehensive nature of our transcriptional analysis is novel in the context of fibrosis, particularly in pulmonary injury triggered by genetic susceptibility of the epithelial compartment and could provide insights in the development of more targeted and effective therapeutics in the future.

To further appreciate the role of the resident alveolar compartment during inflammatory exacerbations progressing to fibrosis, flow cytometry-based sorting of CD11b^-^SigF^+^CD11c^+^ cells was performed 3 days and 14 days after SP-C^I73T^ induced injury. Our results allowed us to place these cells as players of all phases of the SP-C^I73T^ injury, through temporally restricted activation of canonical inflammatory pathways (IL6 and prostaglandin E2 exclusively shown 3 days post injury) and pro-fibrotic pathways (glycoprotein/GP6, SOX2, KLF4). At the same time, we show a subset of signaling networks sustained over our 14 day analysis, albeit driven by distinct transcriptional signatures (IFNγ, TGFβ1, IL-4) (64, 65). While these signals have been previously reported in the fibrotic lung, specific annotation in resident alveolar macrophages strengthens the notion that this population functions as a pleiotropic effector of inflammation and tissue remodeling (66, 67), and further substantiates our prior histopathological investigation of the SP-C^I73T^ injury (dysregulated inflammation and increased mortality) following pharmacological depletion of alveolar macrophages with clodronate liposomes (10).

Due to the phenotypic heterogeneity intrinsic of monocytes/macrophages in chronic disease, we built on our previous characterization of monocyte-derived macrophages through single-cell RNA sequencing (10, 22, 68). Albeit a low-resolution clustering analysis, we identified three macrophage (alveolar macrophages, *Trem2^+^*, and *Mertk^+^*), as well as transcriptionally distinct populations of monocytes (classical - *Itgam/cd11b*, *Cx3cr1*; inflammatory - *Ccr2, Ly6c, Lyz2*) (69). Our findings that complement signaling is involved in SP-C^I73T^ injury and fibrosis is consistent with evidence that soluble defense collagens support activation, proliferation, and tissue-repair functions of macrophages (70–72). Alongside these signals, our results define inflammatory monocytes and monocyte-derived moieties (interstitial macrophages) as centrally involved in fibronectin/FN1 and osteopontin/SPP1 signaling in the fibrosing lung, a finding consistent with bleomycin-induced injury (42, 73–78).

Mertk has been used to identify alveolar macrophages due to its role in mediating phagocytosis of apoptotic cells (79). Our annotation of a *Mertk^int^* cluster in control lungs is consistent to their identity as alveolar macrophages. However, identification of a second population of *Mertk^high^* cells appearing 42 days post injury and displaying a unique transcriptional signatures suggest these may be monocyte-derived moieties settling within the lung following the end of the inflammatory exacerbation. Further work needs to establish their identity since our pseudotime analysis did not fully recapitulate their origin.

Mining of a publicly available human IPF dataset (GSE136831, www.ipfcellatlas.com) offered the opportunity to add a translational value to these findings (40). Our analysis identified three macrophage clusters in the IPF lung that generate a transcriptional signature comparable to our murine results (innate immunity, interleukin mediated signaling, fibrogenic processes, and heightened transcriptional and translational control). In particular, this work confirmed the presence of *TREM2^+^* interstitial macrophages (cluster 0) and a population of alveolar macrophages (cluster 1) in late stage IPF (22, 80, 81). Due to the low resolution of the clustering, our findings do show a single cluster co-expressing *MERTK* and *TREM2*. At this stage, it is unclear if such population appears in established fibrosis as clinical literature seldom cites both markers. Independent of the nomenclature, our assessment is consistent with reports showing osteopontin/SPP1, fibronectin/FN1, and TGFβ1 signaling in the fibrotic niche (73, 82–84).

The importance of defining the metabolic signatures accompanying acute and chronic inflammation has great therapeutic potential (85). Our analysis identifies a robust lipid signature in steady-state alveolar macrophages, a notion consistent with their role in surfactant lipid (fluid) recycling and maintenance (86). The changes observed during SP-C^I73T^ injury are consistent with the notion that pro-fibrotic reprogramming requires a metabolic shift towards lipid consumption (87, 88). Therapeutically noteworthy is the transcriptional signature produced by *Mertk^high^* macrophages 42 days post injury, which included *Pparg* and several apolipoprotein receptors. Indeed, there is a large body of evidence showing that engaging this transcription factor is effective in attenuating the fibrotic phenotype (89–91). By comparison, this work leveraged the specificity of the *Apoe* signal originating from *Trem2* ^+^ cells and recent experimental evidence linking this lipoprotein to pro-fibrotic monocyte-derived macrophages during chemical-induced fibrosis (36, 92–94). Our findings that ApoE deletion does not provide overt benefits on *Spp1^+^* cell accumulation in the foci of injury, total inflammation, or survival is somewhat surprising. Confounding elements related to the compensatory effects of a global knock out, or the impact of ApoE deletion on a surfactant impaired system may need further examination. Despite these incongruences, our results support the value of ApoE as a biomarker indicative of the presence of *Spp1* ^+^ and *Fn1^+^* pro-fibrotic macrophages in the lung.

Though comprehensive, any experimental modeling of disease has limitations. While pairing murine sequencing data with human single-cell datasets offers translational value, elements related to disease staging (early inflammatory exacerbation vs. end-stage disease) and heterogeneity of human IPF etiology make this assessment less obvious. Furthermore, it is well established that use of antibody-based sorting introduces bias to the analysis (e.g., CD11b^-^SigF^+^CD11c^+^ in our bulk sequencing, CD45^+^CD68^+^ for spatial analysis). To a lesser degree, clustering analysis of single-cell RNA sequencing data and cell-communication predictive tools introduce bias related to pathway annotation, and therefore any analysis attempting to describe non-canonical signaling (e.g. ApoE signaling in inflammation rather than lipid homeostasis) may not find a fitting match.

Despite any potential drawback, this work comprehensively assesses the spatial and phenotypic distribution of macrophages in pulmonary fibrosis triggered by a fibrogenic mutation in the alveolar epithelial cell restricted gene encoding for the SP-C. Our data finds peri-injury macrophages to produce an extremely active phenotype, while CD68^+^ cells localized within the fibrotic foci appear transcriptionally dormant at the peak of an inflammation exacerbation. Single cell analysis elucidated the intercellular communications occurring in the lung, while identifying *Ccr2^+^Ly6c* ^+^ inflammatory monocytes and *trem2^+^* interstitial macrophages as distinct fibrogenic populations in SP-C^I73T^ induced injury. Furthermore, our work defined distinct lipid signatures among macrophage populations and propose ApoE as a potential biomarker to identify SPP1- and FN1-producing macrophages. Taken together, this work provides an essential framework for the identification (and future targeting) of deleterious macrophage populations in the early and late stages of the fibrogenic process.

## Data availability statement

The datasets presented in this study can be found in online repositories. The names of the repository/repositories and accession number(s) can be found in the article/**Supplementary Material**.

## Ethics statement

The animal study was approved by the Institutional Animal Care and Use Committee, University of Utah. The study was conducted in accordance with the local legislation and institutional requirements.

## Author contributions

PM: Data curation, Formal analysis, Methodology, Resources, Software, Visualization, Writing – original draft, Writing – review & editing. JC: Conceptualization, Data curation, Methodology, Validation, Writing – review & editing. SC: Data curation, Formal analysis, Methodology, Software, Visualization, Writing – review & editing. JN: Data curation, Formal analysis, Methodology, Visualization, Writing – review & editing. QW: Data curation, Methodology, Visualization, Writing – review & editing. TM: Formal analysis, Methodology, Visualization, Writing – review & editing. AV: Conceptualization, Data curation, Formal analysis, Funding acquisition, Investigation, Methodology, Project administration, Resources, Software, Supervision, Validation, Visualization, Writing – original draft, Writing – review & editing.

## References

[1] Cottin V Hirani NA Hotchkin DL Nambiar AM Ogura T Otaola M. Presentation, diagnosis and clinical course of the spectrum of progressive-fibrosing interstitial lung diseases. Eur Respir Rev. (2018) 27:180076. doi: 10.1183/16000617.0076-2018 30578335 PMC9489068

[2] Wijsenbeek M Cottin V. Spectrum of fibrotic lung diseases. N Engl J Med. (2020) 383:958–68. doi: 10.1056/NEJMra2005230 32877584

[3] Conti S Harari S Caminati A Zanobetti A Schwartz JD Bertazzi PA. The association between air pollution and the incidence of idiopathic pulmonary fibrosis in Northern Italy. Eur Respir J. (2018) 51(1):1700397. doi: 10.1183/13993003.00397-2017 29371377

[4] Winterbottom CJ Shah RJ Patterson KC Kreider ME Panettieri RA Jr. Rivera-Lebron B. Exposure to ambient particulate matter Is associated with accelerated functional decline in idiopathic pulmonary fibrosis. Chest. (2018) 153:1221–8. doi: 10.1016/j.chest.2017.07.034 PMC6026290 28802694

[5] Spagnolo P Kropski JA Jones MG Lee JS Rossi G Karampitsakos T. Idiopathic pulmonary fibrosis: Disease mechanisms and drug development. Pharmacol Ther. (2021) 222:107798. doi: 10.1016/j.pharmthera.2020.107798 33359599 PMC8142468

[6] Cui F Sun Y Xie J Li D Wu M Song L. Air pollutants, genetic susceptibility and risk of incident idiopathic pulmonary fibrosis. Eur Respir J. (2023) 61(2):2200777. doi: 10.1183/13993003.00777-2022 36137588

[7] Sesé L Harari S. Now we know: chronic exposure to air pollutants is a risk factor for the development of idiopathic pulmonary fibrosis. Eur Respir J. (2023) 61(2):2202113. doi: 10.1183/13993003.02113-2022 36731901

[8] Mulugeta S Nureki S Beers MF. Lost after translation: insights from pulmonary surfactant for understanding the role of alveolar epithelial dysfunction and cellular quality control in fibrotic lung disease. Am J Physiol Lung Cell Mol Physiol. (2015) 309:L507–25. doi: 10.1152/ajplung.00139.2015 PMC4572416 26186947

[9] Venosa A Katzen J Tomer Y Kopp M Jamil S Russo SJ. Epithelial expression of an interstitial lung disease–associated mutation in surfactant protein-C modulates recruitment and activation of key myeloid cell populations in mice. J Immunol. (2019) 202:2760–71. doi: 10.4049/jimmunol.1900039 PMC6478557 30910861

[10] Venosa A Cowman S Katzen J Tomer Y Armstrong BS Mulugeta S. Role of CCR2(+) myeloid cells in inflammation responses driven by expression of a surfactant protein-C mutant in the alveolar epithelium. Front Immunol. (2021) 12:665818–8. doi: 10.3389/fimmu.2021.665818 PMC8101410 33968067

[11] Hawkins A Guttentag SH Deterding R Funkhouser WK Goralski JL Chatterjee S. A non-BRICHOS SFTPC mutant (SP-C(I73T)) linked to interstitial lung disease promotes a late block in macroautophagy disrupting cellular proteostasis and mitophagy. Am J Physiol Lung Cell Mol Physiol. (2015) 308:L33–47. doi: 10.1152/ajplung.00217.2014 PMC4281696 25344067

[12] Katzen J Wagner BD Venosa A Kopp M Tomer Y Russo SJ. An SFTPC BRICHOS mutant links epithelial ER stress and spontaneous lung fibrosis. JCI Insight. (2019) 4(6):e126125. doi: 10.1172/jci.insight.126125 30721158 PMC6483196

[13] Teoh AKY Jo HE Chambers DC Symons K Walters EH Goh NS. Blood monocyte counts as a potential prognostic marker for idiopathic pulmonary fibrosis: analysis from the Australian IPF registry. Eur Respir J. (2020) 55(4):1901855. doi: 10.1183/13993003.01855-2019 31949112

[14] Kreuter M Lee JS Tzouvelekis A Oldham JM Molyneaux PL Weycker D. Monocyte count as a prognostic biomarker in patients with idiopathic pulmonary fibrosis. Am J Respir Crit Care Med. (2021) 204:74–81. doi: 10.1164/rccm.202003-0669OC 33434107 PMC8437112

[15] Gibson CD Kugler MC Deshwal H Munger JS Condos R. Advances in targeted therapy for progressive fibrosing interstitial lung disease. Lung. (2020) 198:597–608. doi: 10.1007/s00408-020-00370-1 32591895

[16] Janssen WJ Barthel L Muldrow A Oberley-Deegan RE Kearns MT Jakubzick C. Fas determines differential fates of resident and recruited macrophages during resolution of acute lung injury. Am J Respir Crit Care Med. (2011) 184:547–60. doi: 10.1164/rccm.201011-1891OC PMC3175550 21471090

[17] Gibbings SL Goyal R Desch AN Leach SM Prabagar M Atif SM. Transcriptome analysis highlights the conserved difference between embryonic and postnatal-derived alveolar macrophages. Blood. (2015) 126:1357–66. doi: 10.1182/blood-2015-01-624809 PMC4566811 26232173

[18] Tan SY Krasnow MA. Developmental origin of lung macrophage diversity. Development. (2016) 143:1318–27. doi: 10.1242/dev.129122 PMC4852511 26952982

[19] Reyfman PA Walter JM Joshi N Anekalla KR Mcquattie-Pimentel AC Chiu S. Single-cell transcriptomic analysis of human lung provides insights into the pathobiology of pulmonary fibrosis. Am J Respir Crit Care Med. (2019) 199:1517–36. doi: 10.1164/rccm.201712-2410OC PMC6580683 30554520

[20] Joshi N Watanabe S Verma R Jablonski RP Chen C-I Cheresh P. A spatially restricted fibrotic niche in pulmonary fibrosis is sustained by M-CSF/M-CSFR signalling in monocyte-derived alveolar macrophages. Eur Respir J. (2020) 55(1):1900646. doi: 10.1183/13993003.00646-2019 31601718 PMC6962769

[21] Young LR Gulleman PM Short CW Tanjore H Sherrill T Qi A. Epithelial-macrophage interactions determine pulmonary fibrosis susceptibility in Hermansky-Pudlak syndrome. JCI Insight. (2016) 1:e88947. doi: 10.1172/jci.insight.88947 27777976 PMC5070955

[22] Misharin AV Morales-Nebreda L Reyfman PA Cuda CM Walter JM Mcquattie-Pimentel AC. Monocyte-derived alveolar macrophages drive lung fibrosis and persist in the lung over the life span. J Exp Med. (2017) 214:2387–404. doi: 10.1084/jem.20162152 PMC5551573 28694385

[23] Takahashi F Takahashi K Okazaki T Maeda K Ienaga H Maeda M. Role of osteopontin in the pathogenesis of bleomycin-induced pulmonary fibrosis. Am J Respir Cell Mol Biol. (2001) 24:264–71. doi: 10.1165/ajrcmb.24.3.4293 11245625

[24] Dong J Ma Q. Osteopontin enhances multi-walled carbon nanotube-triggered lung fibrosis by promoting TGF-β1 activation and myofibroblast differentiation. Particle Fibre Toxicol. (2017) 14:18. doi: 10.1186/s12989-017-0198-0 PMC5465601 28595626

[25] Upagupta C Shimbori C Alsilmi R Kolb M. Matrix abnormalities in pulmonary fibrosis. Eur Respir Rev. (2018) 27:180033. doi: 10.1183/16000617.0033-2018 29950306 PMC9489108

[26] Gao X Jia G Guttman A Depianto DJ Morshead KB Sun K-H. Osteopontin links myeloid activation and disease progression in systemic sclerosis. Cell Rep Med. (2020) 1:100140. doi: 10.1016/j.xcrm.2020.100140 33294861 PMC7691442

[27] Hatipoglu OF Uctepe E Opoku G Wake H Ikemura K Ohtsuki T. Osteopontin silencing attenuates bleomycin-induced murine pulmonary fibrosis by regulating epithelial–mesenchymal transition. Biomedicine Pharmacotherapy. (2021) 139:111633. doi: 10.1016/j.biopha.2021.111633 34243624

[28] Conroy LR Clarke HA Allison DB Valenca SS Sun Q Hawkinson TR. Spatial metabolomics reveals glycogen as an actionable target for pulmonary fibrosis. Nat Commun. (2023) 14(1):2759. doi: 10.1038/s41467-023-38437-1 37179348 PMC10182559

[29] Rajesh R Atallah R Bärnthaler T. Dysregulation of metabolic pathways in pulmonary fibrosis. Pharmacol Ther. (2023) 246:108436. doi: 10.1016/j.pharmthera.2023.108436 37150402

[30] Kelly B O’neill LA. Metabolic reprogramming in macrophages and dendritic cells in innate immunity. Cell Res. (2015) 25:771–84. doi: 10.1038/cr.2015.68 PMC4493277 26045163

[31] Liu Y Xu R Gu H Zhang E Qu J Cao W. Metabolic reprogramming in macrophage responses. biomark Res. (2021) 9:1. doi: 10.1186/s40364-020-00251-y 33407885 PMC7786975

[32] Wang S Liu G Li Y Pan Y. Metabolic reprogramming induces macrophage polarization in the tumor microenvironment. Front Immunol. (2022) 13:840029. doi: 10.3389/fimmu.2022.840029 35874739 PMC9302576

[33] Dorotea D Koya D Ha H. Recent insights into SREBP as a direct mediator of kidney fibrosis via lipid-independent pathways. Front Pharmacol. (2020) 11. doi: 10.3389/fphar.2020.00265 PMC7092724 32256356

[34] Jaroonwitchawan T Arimochi H Sasaki Y Ishifune C Kondo H Otsuka K. Stimulation of the farnesoid X receptor promotes M2 macrophage polarization. Front Immunol. (2023) 14. doi: 10.3389/fimmu.2023.1065790 PMC9911659 36776885

[35] Baitsch D Bock HH Engel T Telgmann R Muller-Tidow C Varga G. Apolipoprotein E induces antiinflammatory phenotype in macrophages. Arterioscler Thromb Vasc Biol. (2011) 31:1160–8. doi: 10.1161/ATVBAHA.111.222745 PMC3529398 21350196

[36] Cui H Jiang D Banerjee S Xie N Kulkarni T Liu RM. Monocyte-derived alveolar macrophage apolipoprotein E participates in pulmonary fibrosis resolution. JCI Insight. (2020) 5(5):e134539. doi: 10.1172/jci.insight.134539 32027623 PMC7141408

[37] Venosa A Malaviya R Gow AJ Hall L Laskin JD Laskin DL. Protective role of spleen-derived macrophages in lung inflammation, injury, and fibrosis induced by nitrogen mustard. Am J Physiol Lung Cell Mol Physiol. (2015) 309:L1487–1498. doi: 10.1152/ajplung.00276.2015 PMC4683320 26475734

[38] R Core Team. R: A language and environment for statistical computing. R Foundation for Statistical Computing. Vienna, Austria (2013). Available at: https://www.R-project.org/.

[39] Love MI Huber W Anders S. Moderated estimation of fold change and dispersion for RNA-seq data with DESeq2. Genome Biol. (2014) 15:550. doi: 10.1186/s13059-014-0550-8 25516281 PMC4302049

[40] Adams TS Schupp JC Poli S Ayaub EA Neumark N Ahangari F. Single-cell RNA-seq reveals ectopic and aberrant lung-resident cell populations in idiopathic pulmonary fibrosis. Sci Adv. (2020) 6:eaba1983. doi: 10.1126/sciadv.aba1983 32832599 PMC7439502

[41] Zappia L Oshlack A. Clustering trees: a visualization for evaluating clusterings at multiple resolutions. Gigascience. (2018) 7(7):giy083. doi: 10.1093/gigascience/giy083 30010766 PMC6057528

[42] Aran D Looney AP Liu L Wu E Fong V Hsu A. Reference-based analysis of lung single-cell sequencing reveals a transitional profibrotic macrophage. Nat Immunol. (2019) 20:163–72. doi: 10.1038/s41590-018-0276-y PMC6340744 30643263

[43] Hafemeister C Satija R. Normalization and variance stabilization of single-cell RNA-seq data using regularized negative binomial regression. Genome Biol. (2019) 20:296. doi: 10.1186/s13059-019-1874-1 31870423 PMC6927181

[44] Ahlmann-Eltze C Huber W. glmGamPoi: fitting Gamma-Poisson generalized linear models on single cell count data. Bioinformatics. (2021) 36:5701–2. doi: 10.1093/bioinformatics/btaa1009 PMC8023675 33295604

[45] Hao Y Hao S Andersen-Nissen E Mauck WM 3rd Zheng S Butler A. Integrated analysis of multimodal single-cell data. Cell. (2021) 184:3573–3587.e3529. doi: 10.1016/j.cell.2021.04.048 34062119 PMC8238499

[46] Kuleshov MV Jones MR Rouillard AD Fernandez NF Duan Q Wang Z. Enrichr: a comprehensive gene set enrichment analysis web server 2016 update. Nucleic Acids Res. (2016) 44:W90–97. doi: 10.1093/nar/gkw377 PMC4987924 27141961

[47] Trapnell C Cacchiarelli D Grimsby J Pokharel P Li S Morse M. The dynamics and regulators of cell fate decisions are revealed by pseudotemporal ordering of single cells. Nat Biotechnol. (2014) 32:381–6. doi: 10.1038/nbt.2859 PMC4122333 24658644

[48] Qiu X Mao Q Tang Y Wang L Chawla R Pliner HA. Reversed graph embedding resolves complex single-cell trajectories. Nat Methods. (2017) 14:979–82. doi: 10.1038/nmeth.4402 PMC5764547 28825705

[49] Cao J Spielmann M Qiu X Huang X Ibrahim DM Hill AJ. The single-cell transcriptional landscape of mammalian organogenesis. Nature. (2019) 566:496–502. doi: 10.1038/s41586-019-0969-x 30787437 PMC6434952

[50] Dimitrov D Türei D Garrido-Rodriguez M Burmedi PL Nagai JS Boys C. Comparison of methods and resources for cell-cell communication inference from single-cell RNA-Seq data. Nat Commun. (2022) 13(1):3224. doi: 10.1038/s41467-022-30755-0 35680885 PMC9184522

[51] Dimitrov D Schäfer PSL Farr E Mier PR Lobentanzer S Dugourd A. LIANA+: an all-in-one cell-cell communication framework. bioRxiv. (2023) 2023.08.19.553863. doi: 10.1101/2023.08.19.553863

[52] Tu J-J Li H-S Yan H Zhang X-F. EnDecon: cell type deconvolution of spatially resolved transcriptomics data via ensemble learning. Bioinformatics. (2023) 39:btac825. doi: 10.1093/bioinformatics/btac825 36610709 PMC9825263

[53] Jin S Guerrero-Juarez CF Zhang L Chang I Ramos R Kuan C-H. Inference and analysis of cell-cell communication using CellChat. Nat Commun. (2021) 12(1):1088. doi: 10.1038/s41467-021-21246-9 33597522 PMC7889871

[54] Lane-Donovan C Herz J. ApoE, apoE receptors, and the synapse in alzheimer’s disease. Trends Endocrinol Metab. (2017) 28:273–84. doi: 10.1016/j.tem.2016.12.001 PMC5366078 28057414

[55] Kropski JA Blackwell TS Loyd JE. The genetic basis of idiopathic pulmonary fibrosis. Eur Respir J. (2015) 45:1717–27. doi: 10.1183/09031936.00163814 PMC4849867 25837031

[56] Cook DN Brass DM Schwartz DA. A matrix for new ideas in pulmonary fibrosis. Am J Respir Cell Mol Biol. (2002) 27:122–4. doi: 10.1165/ajrcmb.27.2.f245 12151302

[57] Hardie WD Hagood JS Dave V Perl AK Whitsett JA Korfhagen TR. Signaling pathways in the epithelial origins of pulmonary fibrosis. Cell Cycle. (2010) 9:2769–76. doi: 10.4161/cc.9.14.12268 PMC3040960 20676040

[58] Winters NI Burman A Kropski JA Blackwell TS. Epithelial injury and dysfunction in the pathogenesis of idiopathic pulmonaryFibrosis. Am J Med Sci. (2019) 357:374–8. doi: 10.1016/j.amjms.2019.01.010 PMC6481315 31010463

[59] Nureki SI Tomer Y Venosa A Katzen J Russo SJ Jamil S. Expression of mutant Sftpc in murine alveolar epithelia drives spontaneous lung fibrosis. J Clin Invest. (2018) 128:4008–24. doi: 10.1172/JCI99287 PMC6118576 29920187

[60] Ciccimarra R Bolognesi MM Zoboli M Cattoretti G Stellari FF Ravanetti F. The normal and fibrotic mouse lung classified by spatial proteomic analysis. Sci Rep. (2022) 12(1):8742. doi: 10.1038/s41598-022-12738-9 35610327 PMC9130283

[61] Blumhagen RZ Kurche JS Cool CD Walts AD Heinz D Fingerlin TE. Spatially distinct molecular patterns of gene expression in idiopathic pulmonary fibrosis. Respir Res. (2023) 24:287. doi: 10.1186/s12931-023-02572-6 37978501 PMC10655274

[62] Nuovo GJ Hagood JS Magro CM Chin N Kapil R Davis L. The distribution of immunomodulatory cells in the lungs of patients with idiopathic pulmonary fibrosis. Modern Pathol. (2012) 25:416–33. doi: 10.1038/modpathol.2011.166 PMC3270219 22037258

[63] Eyres M Bell JA Davies ER Fabre A Alzetani A Jogai S. Spatially resolved deconvolution of the fibrotic niche in lung fibrosis. Cell Rep. (2022) 40:111230. doi: 10.1016/j.celrep.2022.111230 35977489 PMC10073410

[64] Wu Q Zhang K-J Jiang S-M Fu L Shi Y Tan R-B. p53: A key protein that regulates pulmonary fibrosis. Oxid Med Cell Longevity. (2020) 2020:6635794. doi: 10.1155/2020/6635794 PMC7721501 33312337

[65] Chandran RR Xie Y Gallardo-Vara E Adams T Garcia-Milian R Kabir I. Distinct roles of KLF4 in mesenchymal cell subtypes during lung fibrogenesis. Nat Commun. (2021) 12(1):7179. doi: 10.1038/s41467-021-27499-8 34893592 PMC8664937

[66] Lee C-M Cho SJ Cho W-K Park JW Lee J-H Choi AM. Laminin α1 is a genetic modifier of TGF-β1–stimulated pulmonary fibrosis. JCI Insight. (2018) 3(18):e99574. doi: 10.1172/jci.insight.99574 30232270 PMC6237225

[67] Uhl K Paithankar S Leshchiner D Jager TE Abdelgied M Dixit B. Differential transcriptomic signatures of small airway cell cultures derived from IPF and COVID-19-induced exacerbation of interstitial lung disease. Cells. (2023) 12(20):2501. doi: 10.3390/cells12202501 37887346 PMC10605205

[68] Nguyen J Deering-Rice CE Armstrong BS Massa C Reilly CA Venosa A. Parenchymal and inflammatory cell responses to single and repeated ozone exposure in healthy and surfactant protein-C mutant lung. Toxicol Sci. (2022) 189:107–23. doi: 10.1093/toxsci/kfac074 PMC9412175 35866636

[69] Trzebanski S Jung S. Plasticity of monocyte development and monocyte fates. Immunol Lett. (2020) 227:66–78. doi: 10.1016/j.imlet.2020.07.007 32814154

[70] Minutti CM Jackson-Jones LH García-Fojeda B Knipper JA Sutherland TE Logan N. Local amplifiers of IL-4Rα-mediated macrophage activation promote repair in lung and liver. Science. (2017) 356:1076–80. doi: 10.1126/science.aaj2067 PMC5737834 28495878

[71] Casals C García-Fojeda B Minutti CM. Soluble defense collagens: Sweeping up immune threats. Mol Immunol. (2019) 112:291–304. doi: 10.1016/j.molimm.2019.06.007 31228661

[72] Sikkeland LIB Ueland T Lund MB Durheim MT Mollnes TE. A role for the terminal C5-C9 complement pathway in idiopathic pulmonary fibrosis. Front Med. (2023) 10. doi: 10.3389/fmed.2023.1236495 PMC10444977 37621463

[73] Morse C Tabib T Sembrat J Buschur KL Bittar HT Valenzi E. Proliferating SPP1/MERTK-expressing macrophages in idiopathic pulmonary fibrosis. Eur Respir J. (2019) 54(2):1802441. doi: 10.1183/13993003.02441-2018 31221805 PMC8025672

[74] Matsubara E Komohara Y Esumi S Shinchi Y Ishizuka S Mito R. SPP1 derived from macrophages is associated with a worse clinical course and chemo-resistance in lung adenocarcinoma. Cancers (Basel). (2022) 14(18):4374. doi: 10.3390/cancers14184374 36139536 PMC9496817

[75] Wang J Zhang L Luo L He P Xiong A Jiang M. Characterizing cellular heterogeneity in fibrotic hypersensitivity pneumonitis by single-cell transcriptional analysis. Cell Death Discovery. (2022) 8:38. doi: 10.1038/s41420-022-00831-x 35091537 PMC8795750

[76] Han H Ge X Komakula SSB Desert R Das S Song Z. Macrophage-derived osteopontin (SPP1) protects from nonalcoholic steatohepatitis. Gastroenterology. (2023) 165:201–17. doi: 10.1053/j.gastro.2023.03.228 PMC10986640 37028770

[77] Hoeft K Schaefer GJL Kim H Schumacher D Bleckwehl T Long Q. Platelet-instructed SPP1(+) macrophages drive myofibroblast activation in fibrosis in a CXCL4-dependent manner. Cell Rep. (2023) 42:112131. doi: 10.1016/j.celrep.2023.112131 36807143 PMC9992450

[78] Moore PK Anderson KC Mcmanus SA Tu TH King EM Mould KJ. Single-cell RNA sequencing reveals unique monocyte-derived interstitial macrophage subsets during lipopolysaccharide-induced acute lung inflammation. Am J Physiol Lung Cell Mol Physiol. (2023) 324:L536–l549. doi: 10.1152/ajplung.00223.2022 36852927 PMC10069979

[79] Chakraborty S Singh A Wang L Wang X Sanborn MA Ye Z. Trained immunity of alveolar macrophages enhances injury resolution via KLF4-MERTK-mediated efferocytosis. J Exp Med. (2023) 220(11):e20221388. doi: 10.1084/jem.20221388 37615937 PMC10450795

[80] Kotlyarov S. Participation of ABCA1 transporter in pathogenesis of chronic obstructive pulmonary disease. Int J Mol Sci. (2021) 22(7):3334. doi: 10.3390/ijms22073334 33805156 PMC8037621

[81] Yang HQ Sun H Li K Shao MM Zhai K Tong ZH. Dynamics of host immune responses and a potential function of Trem2(hi) interstitial macrophages in Pneumocystis pneumonia. Respir Res. (2024) 25:72. doi: 10.1186/s12931-024-02709-1 38317180 PMC10845524

[82] Cai B Dongiovanni P Corey KE Wang X Shmarakov IO Zheng Z. Macrophage merTK promotes liver fibrosis in nonalcoholic steatohepatitis. Cell Metab. (2020) 31:406–421.e407. doi: 10.1016/j.cmet.2019.11.013 31839486 PMC7004886

[83] Lv J Gao H Ma J Liu J Tian Y Yang C. Dynamic atlas of immune cells reveals multiple functional features of macrophages associated with progression of pulmonary fibrosis. Front Immunol. (2023) 14. doi: 10.3389/fimmu.2023.1230266 PMC10525351 37771586

[84] She Y Xu X Yu Q Yang X He J Tang XX. Elevated expression of macrophage MERTK exhibits profibrotic effects and results in defective regulation of efferocytosis function in pulmonary fibrosis. Respir Res. (2023) 24:118. doi: 10.1186/s12931-023-02424-3 37120511 PMC10148433

[85] Li J Zhai X Sun X Cao S Yuan Q Wang J. Metabolic reprogramming of pulmonary fibrosis. Front Pharmacol. (2022) 13:1031890. doi: 10.3389/fphar.2022.1031890 36452229 PMC9702072

[86] Poelma DL Ju MR Bakker SC Zimmermann LJ Lachmann BF Van Iwaarden JF. A common pathway for the uptake of surfactant lipids by alveolar cells. Am J Respir Cell Mol Biol. (2004) 30:751–8. doi: 10.1165/rcmb.2003-0127OC 14644915

[87] Wculek SK Dunphy G Heras-Murillo I Mastrangelo A Sancho D. Metabolism of tissue macrophages in homeostasis and pathology. Cell Mol Immunol. (2022) 19:384–408. doi: 10.1038/s41423-021-00791-9 34876704 PMC8891297

[88] Vassiliou E Farias-Pereira R. Impact of lipid metabolism on macrophage polarization: implications for inflammation and tumor immunity. Int J Mol Sci. (2023) 24(15):12032. doi: 10.3390/ijms241512032 37569407 PMC10418847

[89] Chandran S Schilke RM Blackburn CMR Yurochko A Mirza R Scott RS. Lipin-1 contributes to IL-4 mediated macrophage polarization. Front Immunol. (2020) 11. doi: 10.3389/fimmu.2020.00787 PMC7214697 32431707

[90] Zhou F Fan X Miao Y. LPIN1 promotes triglycerides synthesis and is transcriptionally regulated by PPARG in buffalo mammary epithelial cells. Sci Rep. (2022) 12(1):2390. doi: 10.1038/s41598-022-06114-w 35149744 PMC8837653

[91] Zeng Q Zhou T-T Huang W-J Huang X-T Huang L Zhang X-H. Asarinin attenuates bleomycin-induced pulmonary fibrosis by activating PPARγ. Sci Rep. (2023) 13:14706. doi: 10.1038/s41598-023-41933-5 37679587 PMC10485066

[92] Venosa A Malaviya R Choi H Gow AJ Laskin JD Laskin DL. Characterization of distinct macrophage subpopulations during nitrogen mustard-induced lung injury and fibrosis. Am J Respir Cell Mol Biol. (2016) 54:436–46. doi: 10.1165/rcmb.2015-0120OC PMC4821033 26273949

[93] Gordon EM Yao X Xu H Karkowsky W Kaler M Kalchiem-Dekel O. Apolipoprotein E is a concentration-dependent pulmonary danger signal that activates the NLRP3 inflammasome and IL-1β secretion by bronchoalveolar fluid macrophages from asthmatic subjects. J Allergy Clin Immunol. (2019) 144:426–441.e423. doi: 10.1016/j.jaci.2019.02.027 30872118 PMC9152878

[94] Venosa A Smith LC Murray A Banota T Gow AJ Laskin JD. Regulation of macrophage foam cell formation during nitrogen mustard (NM)-induced pulmonary fibrosis by lung lipids. Toxicol Sci. (2019) 172:344–58. doi: 10.1093/toxsci/kfz187 PMC6876262 31428777

