# Supplementary material for: Spatial and phenotypic heterogeneity of resident and monocyte-derived macrophages during inflammatory exacerbations leading to pulmonary fibrosis
Source: Front Immunol. 2024 Jul 19;15:1425466. doi: 10.3389/fimmu.2024.1425466 (PMC11294112; doi:10.3389/fimmu.2024.1425466)
Supplement: Supplementary file 1 [file DataSheet_1.pdf]

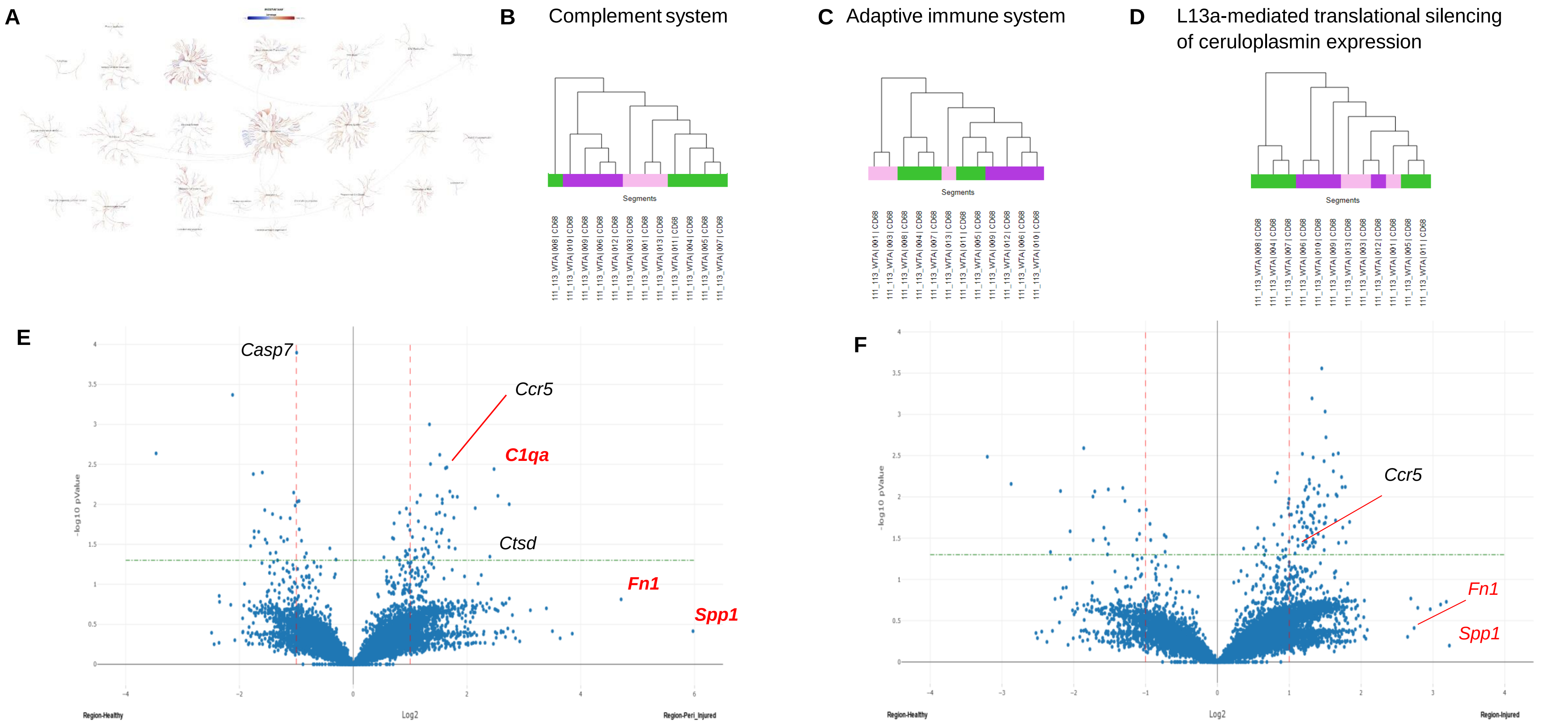

Supp. Figure 1. Pathway analysis of $CD68^+$ macrophages in SP-C^{I73T} induced injury. (**A**) Pathway map of $CD45^+CD68^+$ macrophages samples from controls or 14 days post SP-C^{I73T} injury. **(B-D)** Pathway volcano plots comparison between (B-D) Hierarchical clustered heatmaps depicting the expression of genes associated with 'Complement system', 'Adaptive immune system', and 'L13a-mediated translational silencing of ceruloplasmin expression' in healthy (dark purple), peri-injured (pink), and injured (green) regions of the lung 14 days after SP-C^{I73T} induced injury. (**E-F**) Volcano plot comparing gene expression between (**E**) healthy and peri-injured; (**F**) healthy and injured macrophages. Fold changes are represented on log2 scale. Significance is shown as -log10(pvalue) using linear mixed model. In red are representative complement and fibrosis-associated genes.

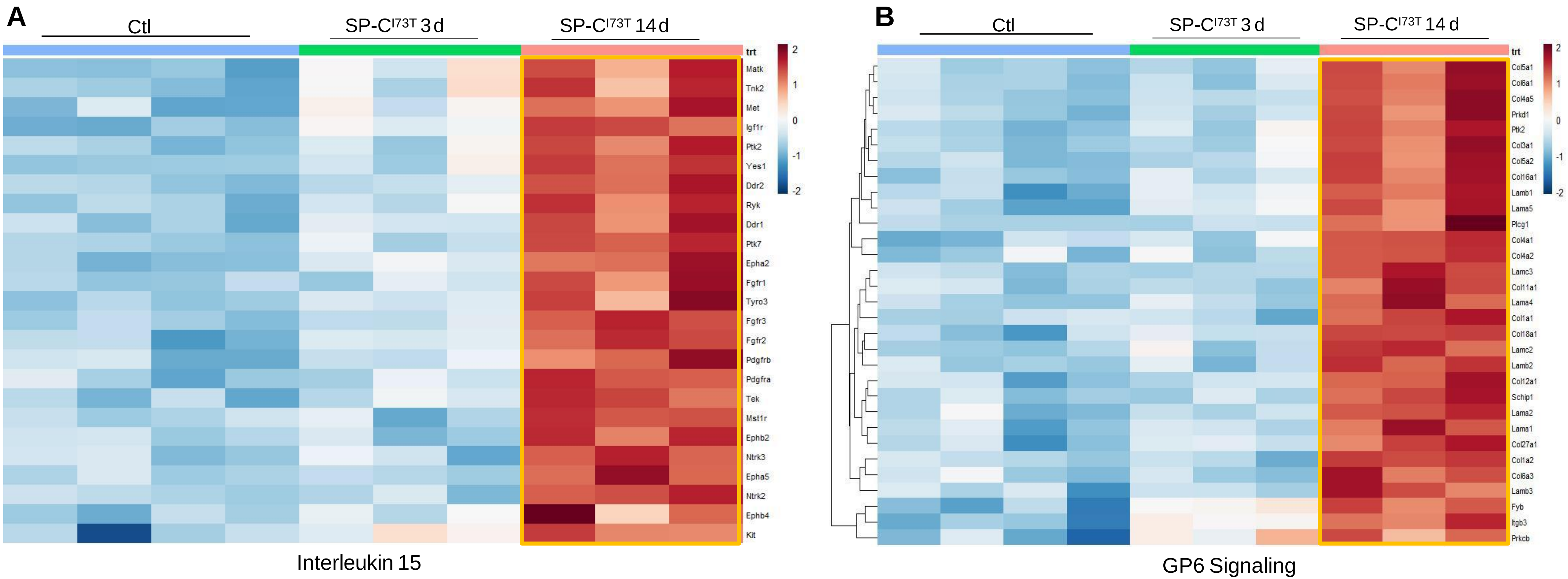

Supp. Figure 2. Transcriptional analysis of resident alveolar macrophages following SP-C^{I73T} induced injury. Bulk RNA sequencing of flow cytometry sorted CD11b^{-}SigF^{+}CD11c^{+}CD64^{+} resident lung macrophages from control (N=4; Ctl, oil treated SP-C^{I73T} mice) or tamoxifen treated SP-C^{I73T} mice at 3 d and 14 d (N=3 for each condition). Heat maps depicting significantly altered genes associated with **(A)** Interleukin 15 and **(B)** GP6 signaling 3- and 14-days after injury relative to Ctl; criteria for significance was a 5% false discovery rate. Note orange and black boxes highlight signatures specific to a given time point.

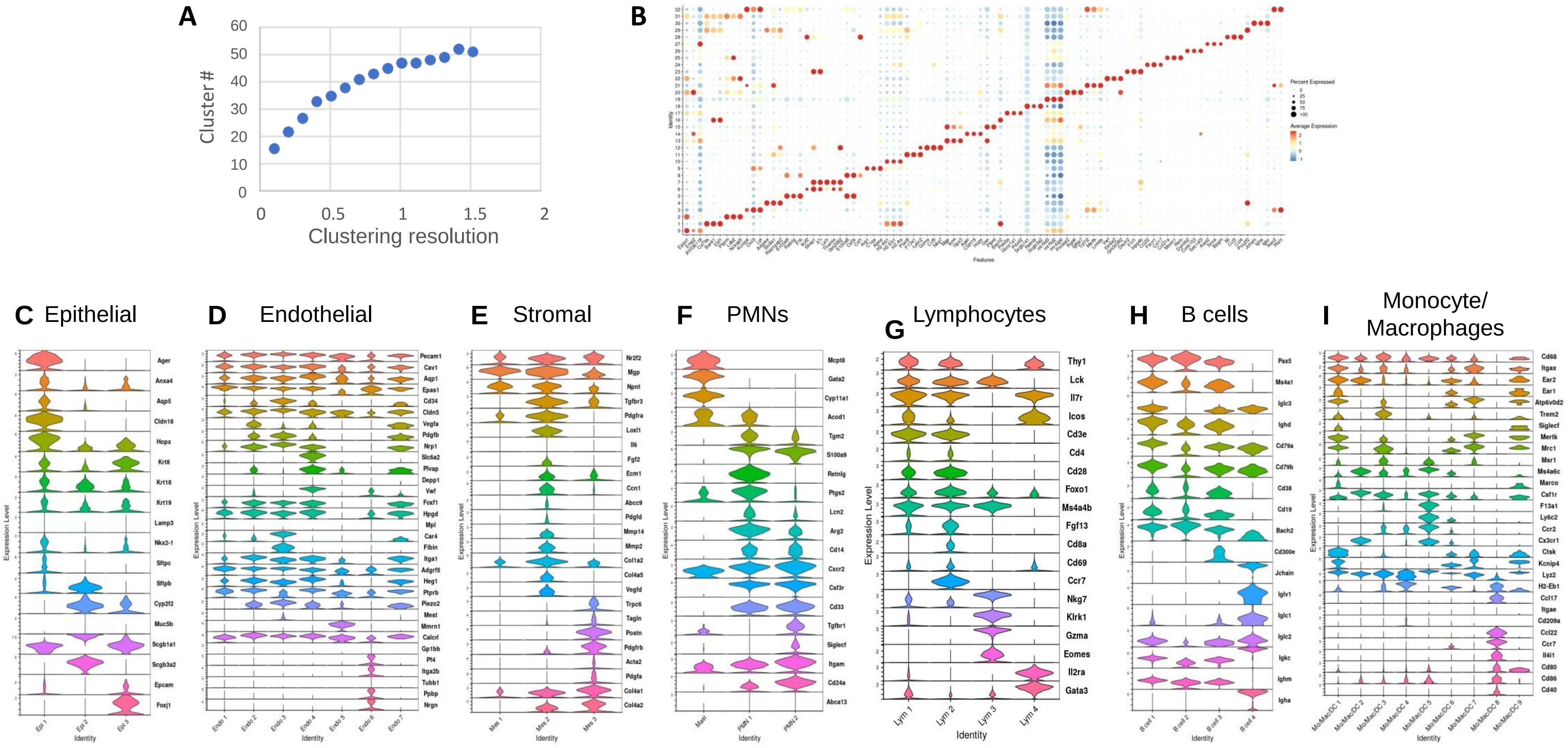

Supp. Figure 3. Single-cell RNA sequencing analysis in the SP-C^{I73T} lung. (A) Cartesian plot depicting number of annotated clusters (y-axis) and clustering resolution (x-axis). Analysis was performed at 0.4 resolution. (**B**) Top 3 non redundant genes expressed by the 33 resulting clusters. (**C-I**) Violin plot depicting expression of a manually curated gene set of established identity markers for (**C**) alveolar epithelial type 1 and type 2 (Epi1), distal airway cells epithelial (Epi2), proximal airway and ciliated cells (Epi3); (**D**) endothelial (capillaries, arteries, veins, lymphatics, megakaryocytes); (**E**) mesenchymal/stromal (Mes/stromal 1 – *Mgp, Mpnt, Nr2f2*; Mes/stromal 2 – *Ecm1, Col1a1, Pdgfr1, Ccn1*; Mes/stromal 3 – *Acta2, Postn, Trpc6, Col4a1*); (**F**) mast cells and polymorphonuclear cells (PMN 1 – neutrophils – *Ptgs2, Retnlg, S100a9;* PMN 2 – eosinophils – *Tgfbr1, Siglecf, Itgam, Cd24a;* mast cells – *Gata2, Cyp1a1, Mcpt8*); (**G**) lymphocytes (Lym 1 – *Cd3e, Cd28, Icos, Thy1*; Lym 2 – Cd8 T cells – *Cd8a, Cd69, Il7r, Ccr7*; Lym 3 – NK cells – *Nkg7, Klrk1, Gzma*; Lym 4 – *Gata3, Il2ra, Icos, Cd69*); (**H**) B cells (B cell 1 - *Pax5, Ms4a1, Iglc3, Ighd, H2-aa, Cd79a, Cd38, Cd19*; B cell 2 – *Bach2*; B cell 3 – *Cd300e*; B cell 4 - *Jchain, Iglv1, Iglc1, Iglc2, Igkc, Ighm, Igha)*; and (**I**) alveolar macrophages, macrophages, and dendritic cells (Mo/Mac/DC1 -) collected from controls (Ctl, oil treated SP-C^{I73T} mice), 14 days or 42 days post SP-C^{I73T} induction.

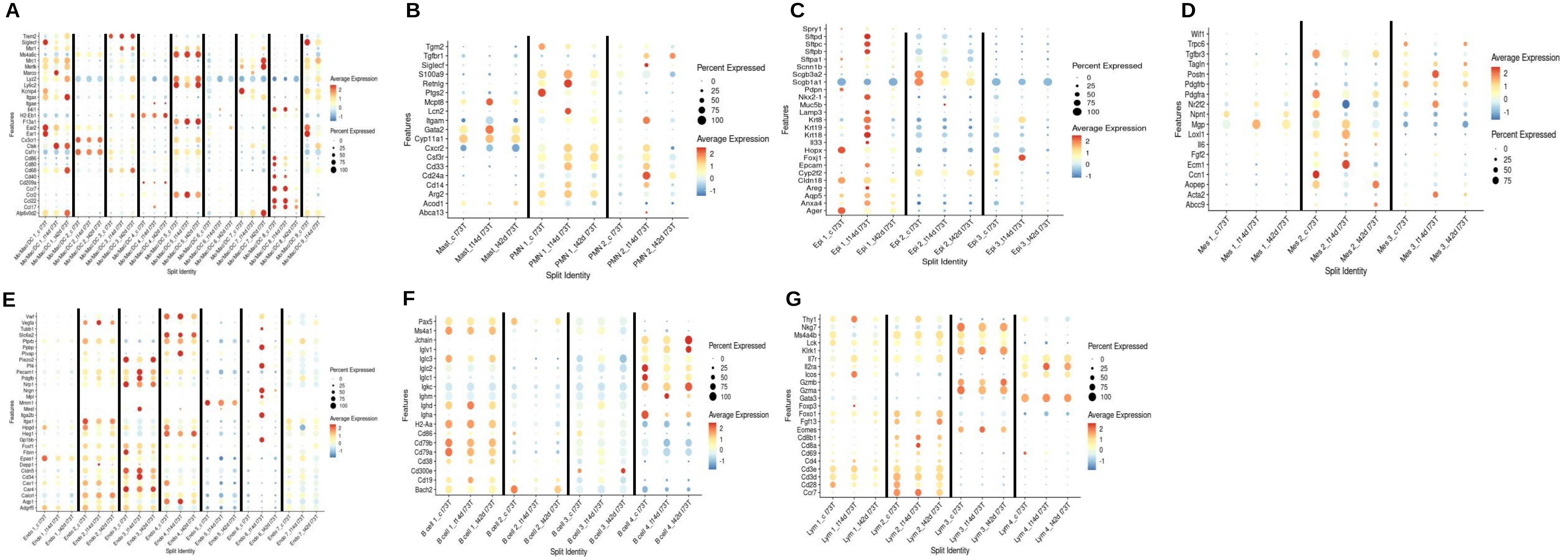

Supp. Figure 4. Split identity analysis of single-cell RNA sequencing dataset collected from the SP-C^{I73T} lung. Split identity bubble plot of identity and activation genes curated for analysis of (**A**) monocytes/macrophages/DCs as listed in the original SingleR/manual annotation, (**B**) mast cells and polymorphonuclear, (**C**) epithelial, (**D**) mesenchymal/stromal cells , (**E**) endothelial, (**F**) B cells, and (**G**) lymphocytes from controls (cI73T), 14 day (t14d I73T), and 42 day (t42d I73T) post SP-C^{I73T} induction.

Macs_Trem2

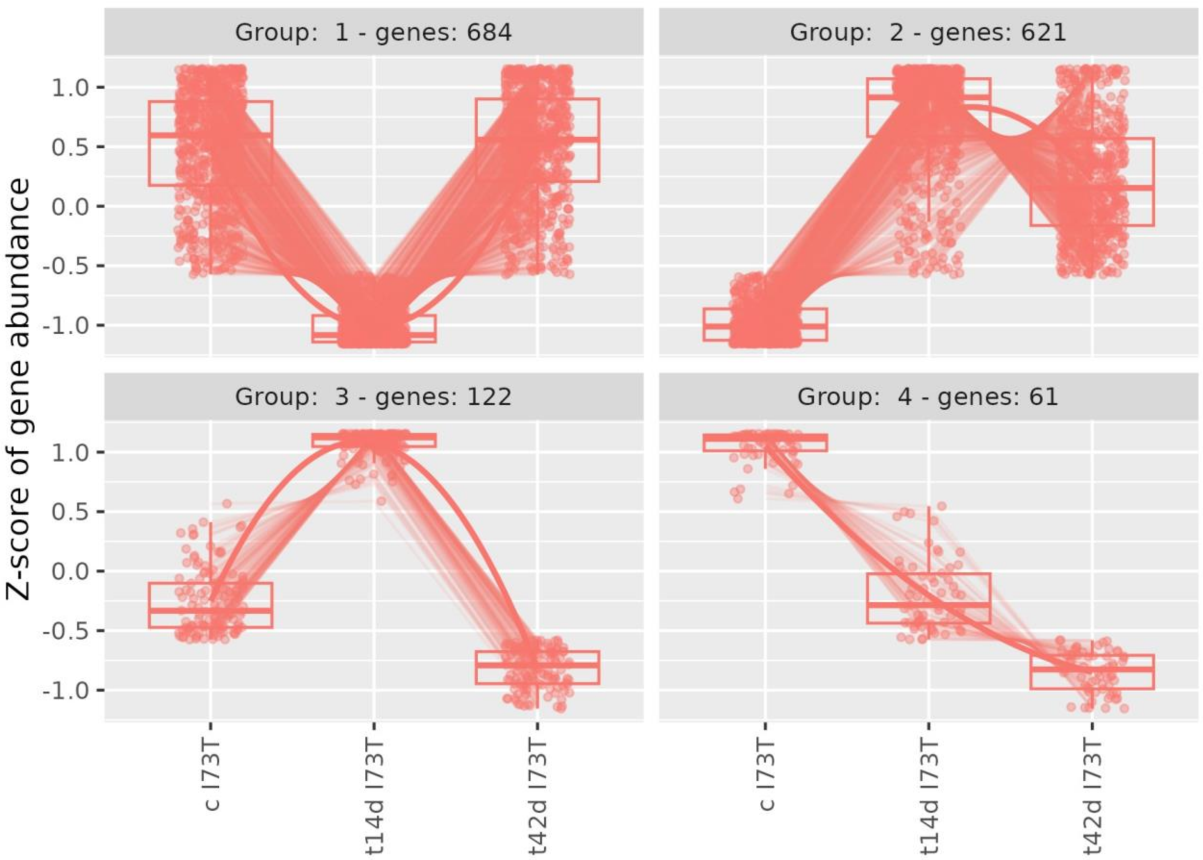

Supp. Figure 5. Pseudobulk analysis of Trem2+ macrophages during SP-C^{I73T} injury. Z-score of gene abundance in Trem2+ macrophages at steady state conditions, 14 days or 42 days post SP-CI73T injury. Four gene expression patterns (groups) are shown.

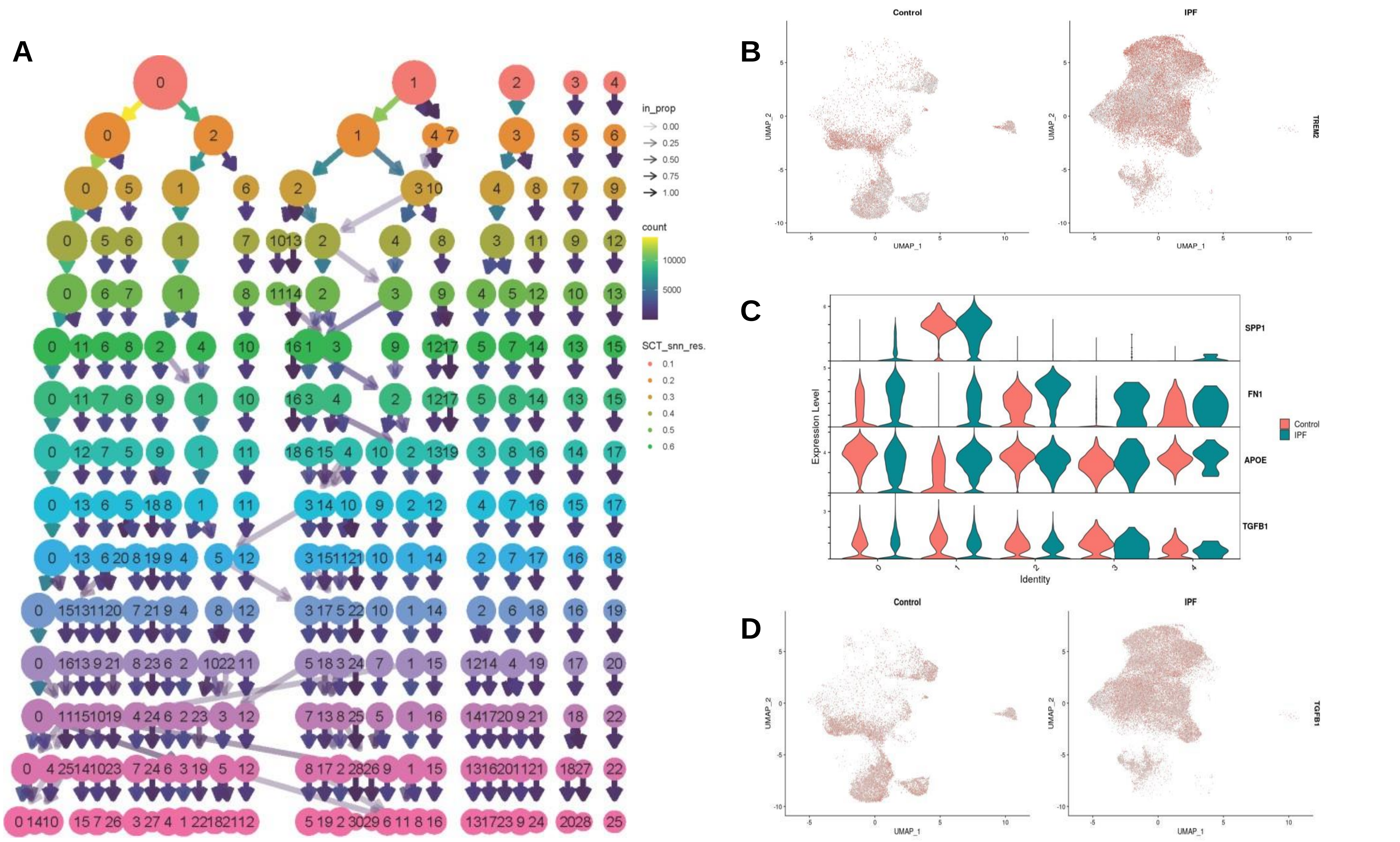

Supp. Figure 6. Single-cell RNA sequencing analysis in the healthy and IPF (human) lung. (A) Clustering tree for dataset GSE136831 depicting proportion and size of annotated clusters at resolutions varying from 0.1 to 1.5. Analysis was performed at 0.1 resolution. (**B**) Split identity UMAP showing expression distribution for *TREM2* in control and IPF lungs. (**C**) Violin plots depicting expression levels for *SPP1, FN1, APOE*, and *TGFB1* in each of the 5 annotated clusters. Note pink color identifying controls, and blue identifying IPF. (**D**) Split identity UMAP showing expression distribution for *TGFB1* in control and IPF lungs.

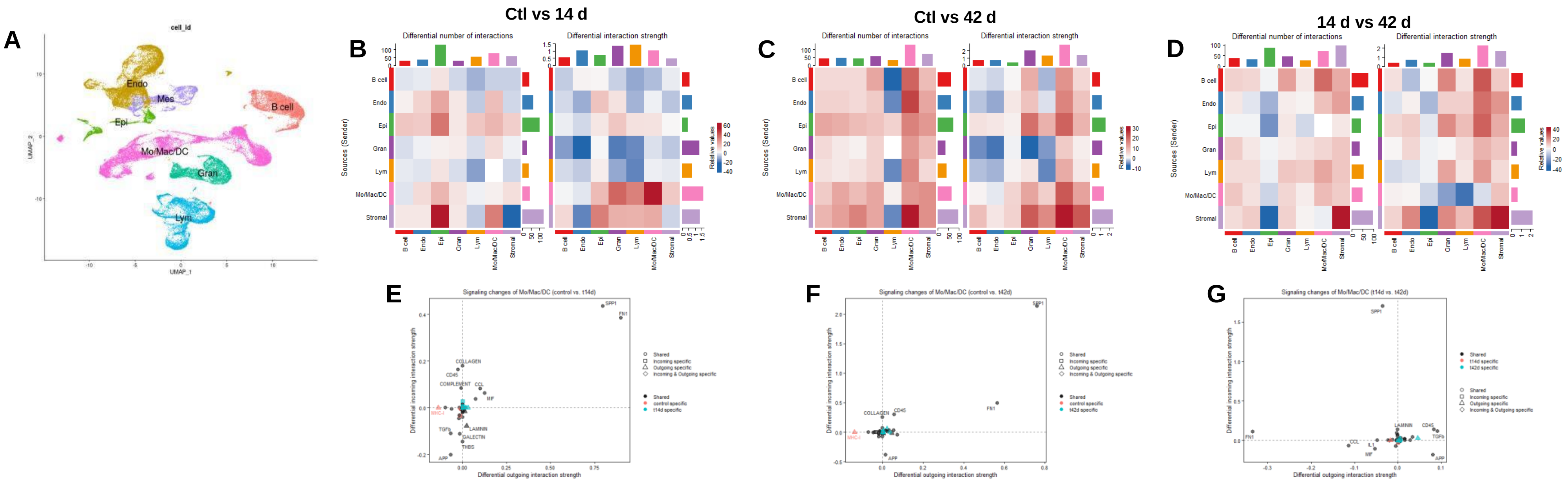

Supp. Figure 7. Cell-cell communication analysis in the SP-C^{I73T} lung. (A) UMAP depiction of cell distribution among “macro-clusters” (epithelial, endothelial, stromal, B cells, granulocytes, lymphocytes, and Mo/Mac/DCs). (**B-D**) CellChat based pairwise comparison examining ligand/sender (y-axis) and receptor/receiver (x-axis) signals between (**B**) controls and 14 days, (**C**) controls and 42 days, and (**D**) 14 days and 42 days. **(E-G)** Prediction of differential signaling pathways in the monocyte/macrophage/DC macro-cluster between (**E**) controls and 14 days, (**F**) controls and 42 days, and (**G**) 14 days and 42 days datasets. Plot legend describes directionality (circle - shared, square – incoming specific, triangle – outgoing specific, diamond – incoming and outgoing specific) and specificity of the signal (black - shared, orange – control specific, cyan – injury/tamoxifen specific).

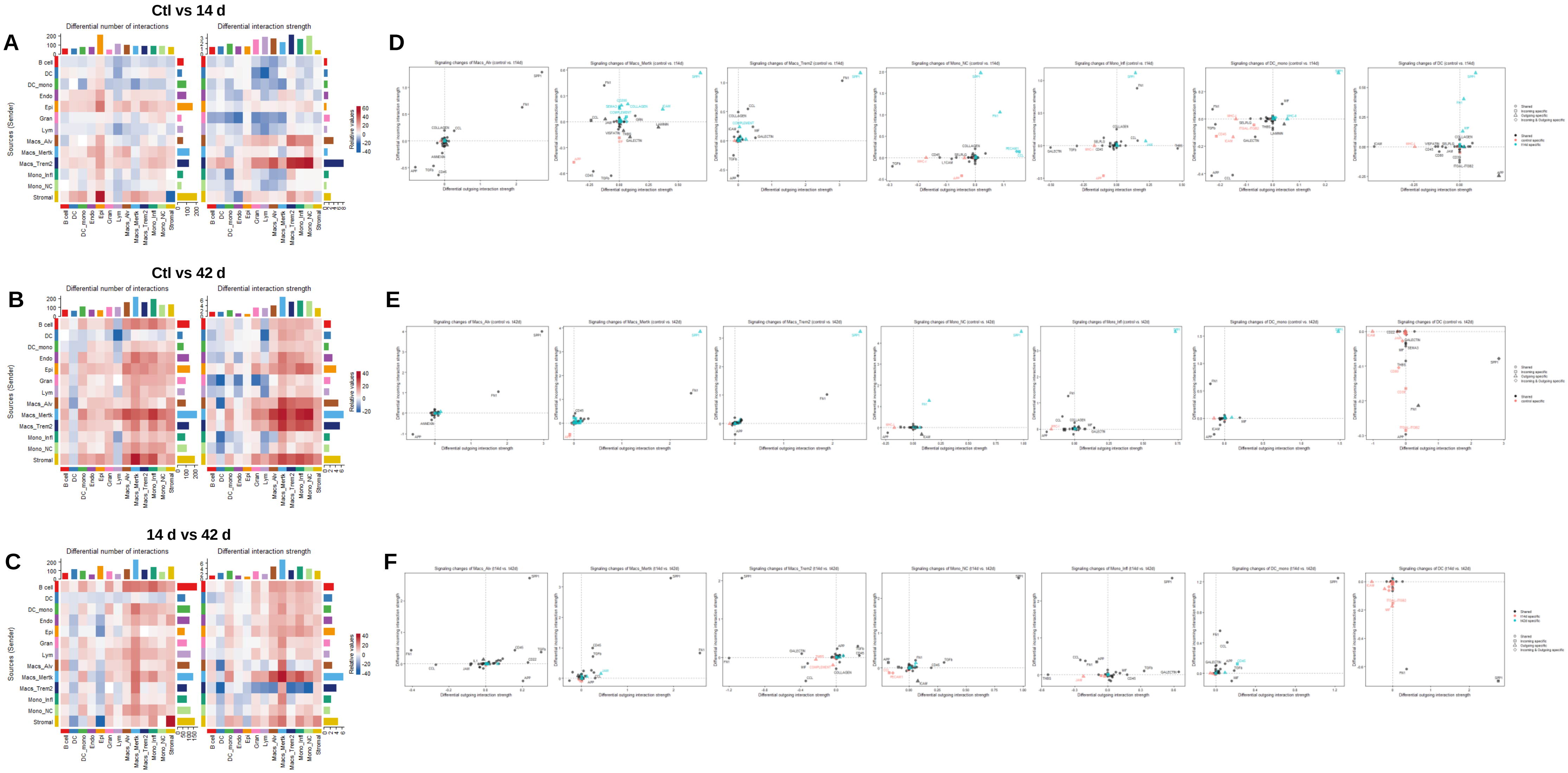

Supp. Figure 8. Cell-cell communication analysis in individual monocyte and macrophage clusters during SP-C^{I73T} injury. (A-C) CellChat based pairwise comparison examining ligand/sender (y-axis) and receptor/receiver (x-axis) signals between (**B**) controls and 14 days, (**C**) controls and 42 days, and (**D**) 14 days and 42 days. Note that monocyte/macrophage/DC macro-cluster was split into: DC, monocyte-derived DC, alveolar macrophages, *Trem2*$^{+}$ macrophages, *Mertk*$^{+}$ macrophages, classical and inflammatory monocytes. **(E-G)** Prediction of differential signaling pathways in the monocyte/macrophage/DC macro-cluster between (**E**) controls and 14 days, (**F**) controls and 42 days, and (**G**) 14 days and 42 days datasets. Plot legend describes directionality (circle - shared, square – incoming specific, triangle – outgoing specific, diamond – incoming and outgoing specific) and specificity of the signal (black - shared, orange – control specific, cyan – injury/tamoxifen specific).
